# The ‘microbiome counterattack’: Insights on the soil and root‐associated microbiome in diverse chickpea and lentil genotypes after an erratic rainfall event

**DOI:** 10.1111/1758-2229.13167

**Published:** 2023-05-24

**Authors:** Francesca Brescia, Fabiano Sillo, Elisabetta Franchi, Ilaria Pietrini, Vincenzo Montesano, Giovanni Marino, Matthew Haworth, Elisa Zampieri, Danilo Fusini, Martino Schillaci, Roberto Papa, Chiara Santamarina, Federico Vita, Walter Chitarra, Luca Nerva, Giannantonio Petruzzelli, Carmelo Mennone, Mauro Centritto, Raffaella Balestrini

**Affiliations:** ^1^ Institute for Sustainable Plant Protection National Research Council of Italy Turin Italy; ^2^ Eni S.p.A. R&D Environmental & Biological Laboratories San Donato Milanese Italy; ^3^ Institute for Sustainable Plant Protection National Research Council of Italy Bernalda (MT) Italy; ^4^ Institute for Sustainable Plant Protection National Research Council of Italy Sesto Fiorentino Italy; ^5^ Department of Agricultural, Food and Environmental Sciences Polytechnic University of Marche Ancona Italy; ^6^ Department of Bioscience, Biotechnology and Environment University of Bari Aldo Moro Bari Italy; ^7^ Research Centre for Viticulture and Enology Council for Agricultural Research and Economics Conegliano Italy; ^8^ Institute of Research on Terrestrial Ecosystems National Research Council of Italy Pisa Italy; ^9^ Azienda Pantanello, ALSIA Research Center Metapontum Agrobios Bernalda (MT) Italy; ^10^ ENI‐CNR Water Research Center ‘Hypatia of Alexandria’ ALSIA Research Center Metapontum Agrobios Bernalda Italy

## Abstract

Legumes maintain soil fertility thanks to their associated microbiota but are threatened by climate change that causes soil microbial community structural and functional modifications. The core microbiome associated with different chickpea and lentil genotypes was described after an unexpected climatic event. Results showed that chickpea and lentil bulk soil microbiomes varied significantly between two sampling time points, the first immediately after the rainfall and the second 2 weeks later. Rhizobia were associated with the soil of the more productive chickpea genotypes in terms of flower and fruit number. The root‐associated bacteria and fungi were surveyed in lentil genotypes, considering that several parcels showed disease symptoms. The metabarcoding analysis revealed that reads related to fungal pathogens were significantly associated with one lentil genotype. A lentil core prokaryotic community common to all genotypes was identified as well as a genotype‐specific one. A higher number of specific bacterial taxa and an enhanced tolerance to fungal diseases characterized a lentil landrace compared to the commercial varieties. This outcome supported the hypothesis that locally adapted landraces might have a high recruiting efficiency of beneficial soil microbes.

## INTRODUCTION

Food legumes are a crucial element to promote the transition towards a plant‐based diet (Bellucci et al., 2021), which is the most important mechanism to promote food security and to respect the planetary boundaries (Gerten et al., 2020). Indeed, food legumes are important crops due to their nutritional value for human consumption. They are rich in proteins and fibres and provide complex carbohydrates, vitamins, and dietary minerals. Lentil (*Lens culinaris* Medik.) and chickpea (*Cicer arietinum* L.) are annual pulse crops native to the Mediterranean, Middle East regions (Van der Maesen, 1987) and South‐West Asia (Weiss & Zohary, 2011). Lentil is a valuable protein source for humans because it is easily digested and the amount of fat and cholesterol is negligible (Sultana & Ghafoor, 2008). Similarly, chickpea provides proteins and beneficial unsaturated fatty acids (Kaur & Prasad, 2021). Chickpeas and lentils are generally cultivated as rainfed crops in arid or semiarid regions on poor‐quality soils (Muehlbauer et al., 1995; Varshney et al., 2013). Besides providing dietary proteins to about 30% of the human population (Afshin et al., 2014), legumes, including chickpeas and lentils, provide important ecosystem services restoring and maintaining soil fertility through their symbiotic association with nitrogen‐fixing rhizobia strains (Postgate, 1998; Sadowsky & Graham, 1998; Tribouillois et al., 2016). Symbiotic rhizobia strains form root nodules, which are considered relevant structures to provide plants with ammonium (Oldroyd, 2013; Udvardi & Poole, 2013). In terrestrial ecosystems, about 80% of the total biologically fixed N is obtained through the legume‐rhizobia symbiosis (Herridge, 2008). The increase in nitrogen uptake, plant growth, and yield parameters of legume crops are well‐known beneficial outcomes of rhizobia‐legumes symbiosis (Erman et al., 2011). In addition to symbiotic relationships, plant roots are in close association with diverse microbial communities at the soil‐root interface. Soil microbiota provides different functions to the agroecosystem and heavily impacts productivity (Almario et al., 2022; Van der Heijden et al., 2008). The impact of soil microbial community on plant health is clearly visible in the case of disease‐suppressive soils, in which specific groups of microorganisms can inhibit the growth of soilborne plant pathogens and thus protect plants from diseases (Weller et al., 2002). However, the mere presence of beneficial microorganisms cannot guarantee plant protection. The interactions within the microbial community itself should be considered, as well as soil physicochemical characteristics, as these aspects may strongly influence the effectiveness of beneficial strains (de Boer et al., 2007; Postma et al., 2008). The impact of climate change and the associated extreme events must also be considered (Trivedi et al., 2022). There is increasing awareness that climate change is threatening agriculture, including pulse crop production, all over the world. The intensification of extreme weather events, irregular patterns of precipitation, drought, and rising temperatures are challenging agricultural productivity and may cause multiple simultaneous crop failures within regions or globally (Janjua et al., 2010; Kirby et al., 2016; Mahmood et al., 2012; Mehrabi & Ramankutty, 2019; Tigchelaar et al., 2018). Awareness of the structural and functional modifications of the microbial community caused by climate change has been increasing in the past decades (Chourasiya et al., 2021; de Vries et al., 2018, 2020; Drigo et al., 2008; Singh et al., 2010; Wahid et al., 2020; Xue et al., 2016). Acquiring knowledge on the core microbiota composition of agricultural crops is pivotal to facing possible modifications in their functional and structural composition caused by climate change.

The initial aim of this study was to describe the core microbiome of different chickpea and lentil genotypes grown in a field experiment, considering different water irrigation treatments and the impact of water limitation on plant growth as well as on soil bacterial communities. However, at the end of the experiment (i.e., during the first sampling and measurement campaign), a major rainfall event temporarily flooded the experimental field. Therefore, the effects of the irrigation variable could not be completely addressed. Consequently, the goal of this study changed during the experimental progress, and attention was focused on the description of the core microbiome composition associated with different chickpea and lentil genotypes after a major climatic event.

## EXPERIMENTAL PROCEDURES

### Field trial, agronomic, and eco‐physiological measurements

The open field experiment was conducted at the Azienda Pantanello (ALSIA research center), Southern Italy (40°23'031.4” N, 16°47'010.9″ E) using four lentil (Colfiorito, Elsa, Eston, and Itaca) and four chickpea genotypes (An.Ca.1586, Nero Tolve, Pascià, and Sultano) provided by the INCREASE consortium (Bellucci et al., 2021) exploiting the single seed descent derived Intelligent Collections of chickpea (Rocchetti et al., 2020, 2022) and lentil (Guerra‐García et al., 2021). Seeds were sown on 04 June 2021. Four replicates for each chickpea and lentil genotypes were considered (i.e., I, II, III, and IV). Three experimental blocks, separated by 2 m to prevent overlapping of the irrigation treatments, were carried out. An inline drip irrigation system (1.3 L/h; 16 mm inside diameter; 10 m wall thickness; 20 cm spacing) provided three irrigation treatments during plant growth. In the first irrigation treatment (unstressed—RG1), water was supplied as needed to minimize water shortage until 10 days prior to maturity. In the second irrigation treatment (RG2), water was provided to a level of 50% of the unstressed parcels, and, the third irrigation treatment (RG3) provided 25% of the level of irrigation supplied to the unstressed treatment. The irrigation system was designed to ensure full water coverage and uniform distribution. Irrigation was applied to all theses when the root zone of the unstressed treatment had lost 60% of its available moisture (defined as the difference in water storage in the root zone between field capacity and wilting point). Therefore, the amount of water given in the fully watered treatment was intended to refill the root zone to close to field capacity, whilst those of the other treatments remained in deficit. The irrigation was applied whenever the sum of daily ETc, excluding the useful rainfall, was equal to 40% of the maximum available soil water content in the 0–30 cm of soil depth, where most of the roots are expected to grow. The daily ETc was calculated according to the formula ETc = ET0 × Kc, where ET0 is the reference evapotranspiration (Eto) according to Penman–Monteith's equation (Allen et al., 1998) and Kc is the crop coefficient, based on recommendations for legumes production and adjusted for the environmental conditions (Oweis et al., 2004; Zhang et al., 2000 and references therein). Water application was monitored via a water meter and unstressed parcels (32 parcels, block 1) received 58,2 cubic meter (m3); the parcels subjected to the RG2 irrigation treatment (32 parcels, block 2) received 31,7 m3, while, the RG3 treatment (32 parcels, block 3) received 19 m^3^ of irrigation water over the whole experimental parcel surface, including lentil and chickpea (4 replicates x 8 genotypes, for a total of 32 parcels in each block). The used irrigation water was high‐quality groundwater (electrical conductivity [EC], 0.431 dS m^−1^; sodium adsorption rate, 1.1; pH, 7.36). Cation concentrations within this water were: Ca^2+^, 1.7; Mg^2+^, 0.9; Na^+^, 1.7; K^+^, 0.06 mmol L^−1^. The corresponding anion concentrations were: CO_3_^2−^, trace; HCO^3−^, 2.6; SO_4_^2−^, 0.8; and NO^3−^, 1.32 mmol L^−1^ (data from Carmelo Mennone, Azienda Pantanello). Each parcel consisted of 11 rows, each 3‐m‐long and 30 cm apart with 3 cm between sown seeds. All recommended cultural practices for lentil and chickpea production were adopted. Weeding was carried out by hand. Strips of land 1‐m‐wide acted as buffer areas between the parcels.

Ten guarded plants were randomly chosen from each parcel to determine plant height (cm). Days to 50% flowering were recorded at proper time and fruits are reported at physiological maturity when 90% of the pods in a parcel were golden brown.

During the phenological stage corresponding to ‘full blooms to early visible pod’ a non‐destructive measurement of physiological variables was performed by means of steady‐state Licor LI‐600 Porometer/Fluorometer for rapid insight into stomatal conductance (gsw).

Two sampling and measurement campaigns were performed (19 July 2021–24 July 2021 and 02 August 2021–05 August 2021). In July (21 July 2021, first time point—T0) and August (04 August 2021, second time point—T1) quantities of chickpea and lentil fruits and flowers were classified on a scale from 0 to 2, where 0 indicates the absence of fruits or flowers, 1 indicates the presence of one‐two plants with flowers or fruits and 2 indicates that fruit or flowers were present in more than two plants. Later, for each genotype, the fruit and flower index (Fr.I and Fl.I) was calculated as follows:

Fr.I. = Frg/*n*,

Fl.I. = Flg/*n*,

where Frg indicates the sum of the fruit class value for each genotype, Flg indicates the sum of the flower class value for each genotype and *n* indicates the replicate number. Therefore, the fruit and flower index value ranged from 0 to 2, where 1 indicates that fruits and flowers were at least present on average in all samples.

### Soil physical–chemical analyses and climatic data

Soil samples from the lentil and chickpea parcels were collected in July (T0) from the 0 to 15 cm layer, pooled according to the water irrigation treatment (RG1, RG2, and RG3), air‐dried and sieved through a 2 mm sieve before laboratory analysis. Physical–chemical features, such as texture, pH, and conductivity, were measured and soil chemical properties were determined according to standard analytical procedures (Sparks et al., 1996). Particle size distribution (sand, silt, and clay) was determined using the pipette method (Gee & Bauder, 1986). Moreover, soils from three different lentil parcels more affected by temporary flooding in RG1 were additionally collected in August at T1 (Table 1). Climatic data, including rainfall, air temperature (minimum, average, and maximum) and relative humidity (average), wind speed (average), and reference Eto were recorded using a meteorological station during the experimental period (Table S1). Data on the same experimental field in 2020 have been reported by Sillo et al. (2022).

**TABLE 1:** Physical–chemical analysis of chickpea and lentil soil.

Chickpea bulk soil	pH	EC	CEC	Clay	Silt	Sand	P Ass	Ca ex	Mg ex	Na ex	K ex	N	C org
T0		μScm^−1^	cmol ^(+)^kg^−1^	%	%	%	mg kg^−1^	g kg^−1^	g kg^−1^	g kg^−1^	g kg^−1^	%	%
RG1C	7.86	1165	25.90	38.90	37.10	24.00	15.80	4.07	0.63	0.39	0.57	0.15	1.39
RG2C	8.18	1295	21.20	35.90	36.20	27.90	16.10	4.06	0.66	0.44	0.52	0.14	1.28
RG3C	8.50	975	24.30	36.70	33.20	30.10	16.50	4.08	0.67	0.41	0.54	0.14	1.22
Mean	8.18	1145	23.80	37.17	35.50	27.33	16.13	4.07	0.65	0.41	0.54	0.14	1.30
sd	0.32	161	2.39	1.55	2.04	3.09	0.35	0.01	0.02	0.03	0.03	0.01	0.09
se	0.18	93	1.38	0.90	1.18	1.78	0.20	0.01	0.01	0.01	0.01	0.00	0.05

Lentil bulk soil	pH	EC	CEC	Clay	Silt	Sand	P Ass	Ca ex	Mg ex	Na ex	K ex	N	C org
T0		μScm^−1^	cmol ^(+)^kg^−1^	%	%	%	mg kg^−1^	g kg^−1^	g kg^−1^	g kg^−1^	g kg^−1^	%	%
RG1L	8.22	1158	27.40	36.70	34.20	29.10	17.20	4.13	0.63	0.40	0.48	0.14	1.29
RG2L	8.15	1265	22.80	35.50	35.60	28.80	15.80	4.09	0.68	0.35	0.47	0.14	1.16
RG3L	8.01	1180	23.60	34.90	35.90	29.20	17.40	4.09	0.68	0.37	0.50	0.13	1.13
Mean	8.13	1201	24.60	35.70	35.23	29.03	16.80	4.10	0.66	0.37	0.48	0.14	1.19
sd	0.11	57	2.46	0.92	0.91	0.21	0.87	0.02	0.03	0.03	0.02	0.01	0.09
se	0.06	33	1.42	0.53	0.52	0.12	0.50	0.01	0.02	0.01	0.01	0.00	0.05

Lentil bulk soil	pH	EC	CEC	Clay	Silt	Sand	P Ass	Ca ex	Mg ex	Na ex	K ex	N	C org
T1		μScm^−1^	cmol ^(+)^kg^−1^	%	%	%	mg kg^−1^	g kg^−1^	g kg^−1^	g kg^−1^	g kg^−1^	%	%
RG1L (ELSA II)	8.67	787	25.60	38.50	36.60	24.90	14.70	3.89	0.62	0.17	0.49	0.09	1.15
RG1L (COLFIORITO I)	8.53	952	23.80	37.50	36.80	25.70	15.60	3.96	0.66	0.15	0.48	0.12	1.09
RG1L (ITACA I)	8.73	832	18.80	36.50	35.80	27.70	15.30	3.92	0.66	0.15	0.42	0.09	1.05
Mean	8.64*	857*	22.73	37.50	36.40	26.10	15.20	3.92	0.65	0.16*	0.46	0.10	1.10
sd	0.10	85	3.52	1.00	0.53	1.44	0.46	0.04	0.02	0.01	0.04	0.02	0.05
se	0.06	49	2.03	0.58	0.31	0.83	0.26	0.02	0.01	0.01	0.02	0.01	0.03

*Note*: The table shows pH, Electric Conductivity (EC, μScm^−1^), Cation Exchange Capacity (CEC, cmol ^(+)^ kg^−1^), the abundance of Clay, Silt, and Sand (%), the assimilable P (Ass. P, mg kg^−1^), the exchangeable Ca, Mg, Na and K (g kg^−1^), the N and organic C percentage (%) of soils in which chickpea (C) and lentil (L) were grown. Chickpea and lentil bulk soil data referred to the first time point (T0) are reported as well as data of lentil soil referred to the second time point (T1). Mean, standard deviation (sd) and standard error (se) are included, asterisks (*) indicate significantly different means (*t* student test *p*‐value < 0.01).

### Bulk soil metabarcoding: Sampling, DNA extraction, and sequencing

Soils were collected from the four different repetitions for each genotype and in each block (i.e., I, II, III, and IV for a total of 96 soil samples) in the first sampling (T0) while, during the second sampling (T1), soils were collected only from the repetition IV. A Dutch soil auger was used to collect soil cores from ground surface depths of 0–15 cm after that any large debris had been removed with a hand trowel. Regarding the first sampling, four replicates for each genotype were pooled inside a treatment (i.e., RG1, RG2, and RG3) for a total of 24 soil samples (3 water levels x 8 genotypes). The genomic DNA was extracted from about 500 mg of each soil sample through the Fast DNA® Spin Kit for Soil (MP Biomedicals). After the quantification by Qubit® 2.0 fluorometer (Invitrogen), 3 ng of the genomic DNA was amplified using the 16S Metagenomics Kit (Thermo Fischer Scientific). The amplification program was the same used by Sillo et al. (2022). Briefly, an initial denaturation step at 95°C for 10 min was followed by 25 cycles at 95°C per 30 s, 58°C for 30 s, and 72°C for 20 s, a final hold time for 7 min at 72°C and cooling step at 4°C. The subsequent purification of the amplicons, the preparation, and the sequencing of the libraries followed the standard protocols for the Ion GeneStudio S5 Systems (i.e., Ion Chef™ System and Ion GeneStudio S5 Sequencer) provided by the manufacturer. The run was based on the workflow Metagenomics 16S w1.1 handling the Database Curated microSEQ®16S and the reference Library 2013.1. The primers detected both ends to obtain 250 bp sequences. Alignment in Torrent Suite™ Software (version 5.16) was performed using the torrent mapping alignment program. The sequences that occurred only once in the entire dataset were removed, and the representative sequences were defined with a 97% similarity cut‐off in order to generate operational taxonomic units (OTUs). When a reading was mapped to multiple locations, the best mapping score was used. If there was more than one, a random mapping with a quality of zero was used. In the output BAM file reports the percentage of reads which passed all filters (i.e., enrichment, no template, clonal and polyclonal discrimination, % of test fragments, % of adapter dimer, and % of low quality). After classifying the OTU representative sequences, the output was elaborated to obtain a relative abundance (%) of each OTU in the total amounts of the entire sample, to estimate the diversity of the microbial community, and to gain a general understanding of the community structure in soil. Obtained reads from metabarcoding of bulk soil samples were submitted to NCBI Sequence Read Archive (SRA) under BioProject PRJNA932564 and PRJNA932977, for chickpea and lentil, respectively.

### Lentil root metabarcoding: Sampling, DNA extraction, and sequencing

*Lens culinaris* root samples (i.e., soil adhering to roots and roots, from now on will be referred to as roots) were collected (one plant per parcel, two parcels per irrigation treatment, hence six replicates per genotype) for a total of 24 parcels initially subjected to diverse irrigation treatments and then differentially affected by the rain (sampling in August 2021). Samples were weighted to a maximum of 250 mg in sterile 2 mL microcentrifuge tubes. Afterwards, a sterile stainless‐steel bead (Ø 5 mm) was added, and the samples were disrupted in liquid nitrogen with a TissueLyser II (Qiagen) at 30 Hz for 30 s. Total DNA was extracted with the DNeasy Plant mini kit (Qiagen) according to the manufacturer's instructions.

Total DNA was quantified with a Nanodrop 2000 (ThermoScientific) and the 260/280 260/230 absorbance ratios were calculated. DNA integrity was further analysed by running the samples on a 1% agarose electrophoretic gel.

To avoid plant DNA amplification, peptide nucleotide acid (PNA) blocker oligos (Kaneka Eurogentec S.A.), targeted at plant mitochondrial and chloroplast 16S rRNA genes and plant Internal Transcribed Spacer (ITS) region of the rRNA gene, were added to the sequencing reaction. PNA was custom‐designed for *L. culinaris* (LcPNA‐CHLORO: GGCTCAACCCTGGACAG; LcPNA‐MITO: GGCAAGTGTTCTTCGGA; LcPNA‐ITS: CGAGGGCACGTCTGCCTGG) and thermal cycler conditions were set as described by the Illumina protocol (Nerva et al., 2019, 2022). Sequencing was carried out by Bio‐Fab Research Srl.

Metabarcoding data were analysed with QIIME 2 (Bolyen et al., 2019). Sequences were trimmed with cutadapt v3.4., denoised through dada2 v2021.8.0, and assembled into Amplicon Sequence Variants (ASVs). ASVs were used instead of OTUs in order to provide a high level of resolution for in‐depth analysis of lentil root samples since they can provide a detailed picture of the diversity within a single sample, as well as they were well suited for reads obtained from Illumina sequencing (Callahan et al., 2016). Additionally, dada2 is optimized for working on Illumina reads (Callahan et al., 2016) and it has been documented that it seems to perform better with Illumina reads due to the higher accuracy and lower error rates of these data compared to Ion Torrent ones (Salipante et al., 2014).

The SILVA v132 and UNITE v8‐99 databases for bacteria and fungi, respectively, were utilized to train Naive Bayes classifier on ASVs (99% identity) sequences for taxonomic assignment. The output was elaborated to obtain a relative abundance (%) of each ASV in the total amounts of the entire sample. Illumina reads from metabarcoding of lentil root samples was submitted to NCBI SRA under BioProject PRJNA933539 for bacterial and fungal reads, respectively.

The ASV table was used as input for Microbiome Analyst (Chong et al., 2020; Dhariwal et al., 2017) for data visualization and statistical assessment. Data were filtered to remove low‐quality and not informative features by setting the minimum count of the low counter filter at 2 and the prevalence in samples at 10%. Data were then rarefied to the minimum library size, scaled with the total sum scaling method, and any data transformation was performed. Diversity within samples at feature level (alpha diversity) was calculated using the Chao1 index, while diversity among samples at feature level (beta diversity) was calculated by the Jaccard index and reported in a two‐dimensional principal coordinates analysis.

The annotation tools FUNGuild (Nguyen et al., 2016) and FAPROTAX (Louca et al., 2016) were used to ascribe a functional ecological role to the fungi and prokaryotes found in the root samples. The Venn diagrams were drawn with the web‐based tool designed by the University of Gent (https://bioinformatics.psb.ugent.be/webtools/Venn/).

### Statistical analysis

Data were analysed with the statistical software Past 4.09 (Hammer et al., 2001) and R 4.1.2 (R Core Team, 2021; https://www.R-project.org/). After verifying the normal distribution and the homogeneity of variances of the molecular, agronomic, and eco‐physiological data, the ANOVA and Tukey post‐hoc comparisons were used, adopting a probability level of *p*‐value ≤ 0.05. If normal distribution and homogeneity of variances were not confirmed, data were analysed with Kruskal–Wallis test and Dunn's post‐hoc comparison was used (*p*‐value ≤ 0.05). The data of physical–chemical soil properties were analysed with *t* student test (*p*‐value ≤ 0.05). The web‐based tool MicrobiomeAnalyst, described above, performed PERMANOVA to analyse the diversity component (alpha and beta).

## RESULTS

### Soil physical–chemical analyses and climatic data

Results from soil physical–chemical analyses are reported in Table 1. Referring to the sampling at T0, the physical–chemical characteristics of chickpea and lentil bulk soil did not differ significantly. Soil pH was around 8.00 (ranging from 7.86 to 8.50). The soil had a clay‐loam texture and the chemical characteristics for the 0–15 cm layer were 1.24% organic carbon content, and EC of soil 1173 μS/cm on average, which are widely considered to be good values for most crops. The N percentage was homogeneous in all samples (around 0.14%) as well as the assimilable P (around 16 mg Kg^−1^), suggesting an average nutrient availability. A significant increase in soil pH was observed in the three lentil parcels at T1 compared to lentil soils at T0 (*p*‐value: 0.0038). The lentil‐associated soil EC and the exchangeable Na (respectively 857 μS/cm and 0.16 g/Kg) were significantly lower compared to lentil bulk soil at T0 (*p*‐value: 0.0043 and 1.30 × 10^−6^, respectively).

The whole experimental period (from 04 June 2021 to 10August 2021) was characterized by an average relative humidity maximum of 81.91%, reached on 19 July 2021, and a minimum of 36.58% on 01 July 2021 (Figure S1). During the experimental period, seven rainy days occurred, and 45.94 mm of rainfall was registered in total. A major rainfall event of 26.93 mm was recorded on 18 July 2021. The maximum air temperature was 41.07°C, recorded on 29 July 2021, while the minimum was 12.57°C registered on 04 June 2021 (day of the sowing) (Table S1).

### Chickpea and lentil agronomic parameters

**FIGURE 1:**
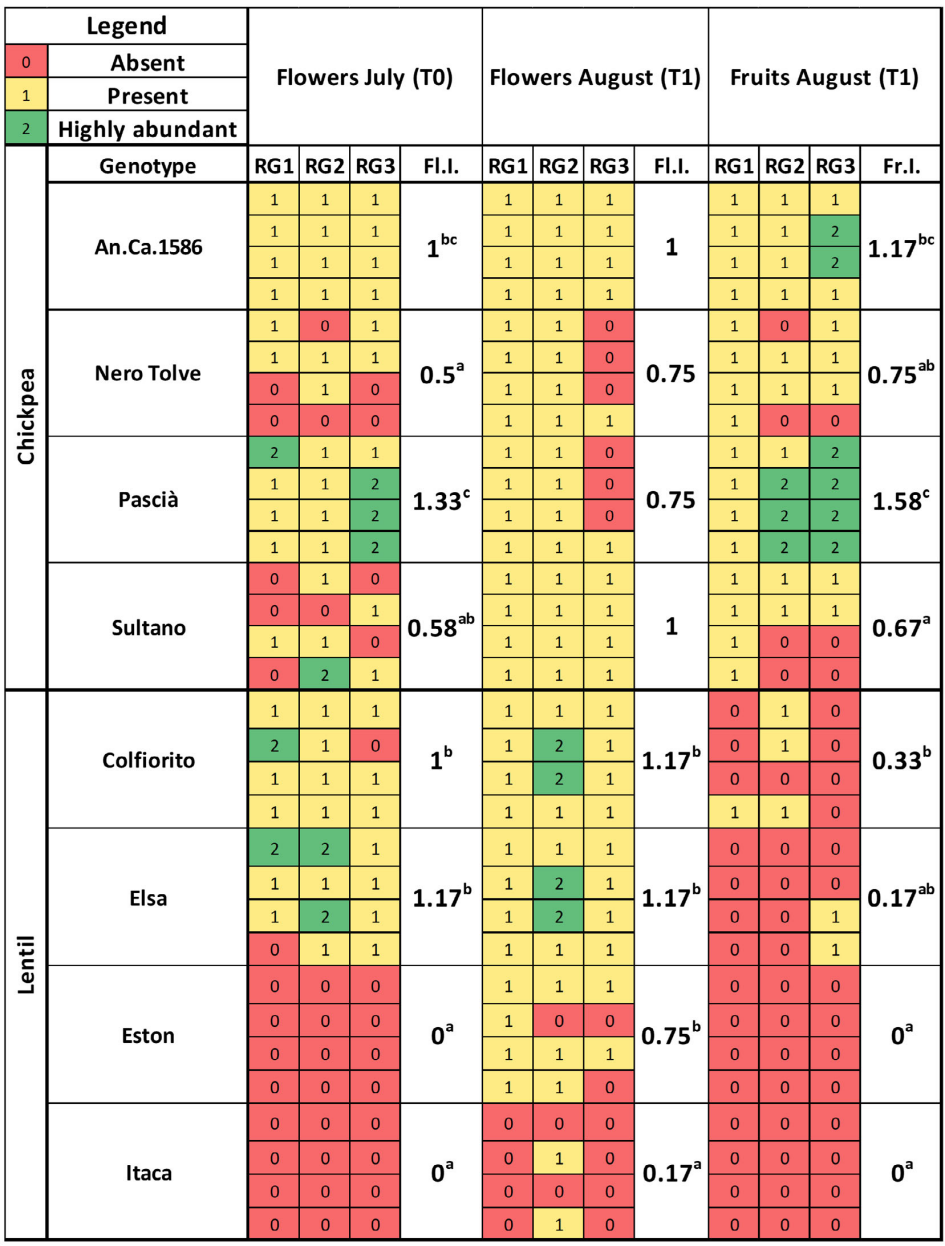
Heatmap of fruit and flower production of chickpea and lentil genotypes in July and August 2021. Chickpea (An.Ca.1586, Nero Tolve, Pascià, Sultano) and lentil (Colfiorito, Elsa, Eston, Itaca) fruit and flower quantities were classified in a scale from 0 to 2 and represented in a heatmap, where 0 (red) indicates absence of fruits or flowers, 1 (yellow) indicates the presence of one‐two plants with flowers or fruits and 2 (green) indicates that fruit or flowers were present in more than two plants. The flower index (Fl.I) and the fruit index (Fr.I.) are reported, different letters indicate significant differences among the genotypes (Kruskal–Wallis test *p*‐value ≤ 0.05, Dunn's post‐hoc comparisons). Irrigation treatments (RG) are reported as 100% water, unstressed (RG1), 50% water (RG2), 25% water (RG3).

Chickpea flowering index in July (T0) was significantly higher in the An.Ca.1586 and Pascià genotypes (1 and 1.33, respectively) compared to Nero Tolve and Sultano (0.50 and 0.58, respectively; Kruskal–Wallis test *p*‐value: 0.0011, Dunn's post‐hoc comparisons). Similarly, chickpea fruit index in August (T1) was significantly higher for the An.Ca.1586 and Pascià genotypes (1.17 and 1.58, respectively), compared to Nero Tolve and Sultano (0.75 and 0.67, respectively). There was no significant difference among chickpea genotypes in the August flowering index, even if the An.Ca.1586 and Sultano genotype flowering indexes (i.e., 1) were slightly higher than Nero Tolve and Pascià (i.e., 0.75) (Figure 1).

Only the Colfiorito and Elsa lentil genotypes had flowers (flowering indices of 1 and 1.17, respectively) in July, while flowers were absent in Eston and Itaca. In August, the Colfiorito, Elsa, and Eston genotypes produced significantly more flowers than Itaca (flowering index of 1.17, 1.17, 0.75, and 0.17, respectively; Kruskal–Wallis test *p*‐value: 9.28 × 10^−6^, Dunn's post‐hoc comparisons). In August, fruit production was exclusively observed in the Colfiorito and Elsa genotypes (fruiting index of 0.33 and 0.17, respectively), while fruits were absent in Eston and Itaca (Figure 1). Indeed, fruit production was significantly higher for Colfiorito compared to the other lentil genotypes (Kruskal–Wallis test *p*‐value: 0.042, Dunn's post‐hoc comparisons; Figure 1).

During the experiment, a major rainfall event occurred (18 July 2021), and the experimental field was flooded. As a result, the irrigation treatments were no longer applied, and the effects of the earlier stress application most likely disappeared. Following the rainfall event, the performed physiological and biometric (i.e., height) measurements did not reveal any significant differences among the genotypes of chickpea and lentil when irrigation treatments were considered (Figures S2 and S3).

### Bulk soil bacterial community

**FIGURE 2:**
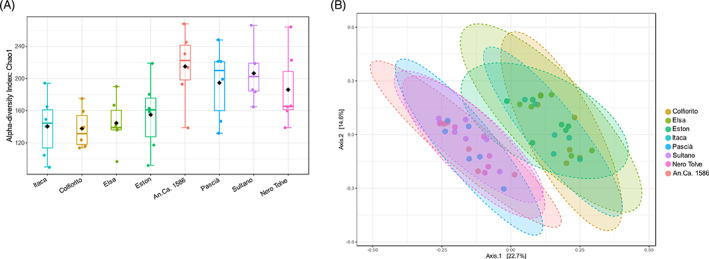
Alpha and beta diversity of chickpea and lentil bulk soil microbiome. The alpha diversity of the four chickpea genotypes (An.Ca.1586, Pascià, Sultano, Nero Tolve) and of the four lentil genotypes (Colfiorito, Elsa, Eston, and Itaca) was evaluated with the Chao1 index and significant differences were evaluated with ANOVA (A). The beta diversity among the four chickpea and four lentil genotypes (B) was evaluated with the Jaccard index and significant differences were evaluated with PERMANOVA. The web‐based tool Microbiome Analyst was used (Dhariwal et al., 2017).

Analysis of the chickpea and lentil bulk soil prokaryotic community revealed that alpha diversity was significantly higher in the chickpea genotypes (*p*‐value: 0.0028052; [ANOVA] *F*‐value: 3.8358; Figure 2). The chickpea and lentil genotypes clustered separately in the beta diversity analysis, indicating that a different species composition characterized the two crop species ([PERMANOVA] *F*‐value: 2.499; R‐squared: 0.30426; *p*‐value: 0.001).

### Chickpea bulk soil bacterial community

**FIGURE 3:**
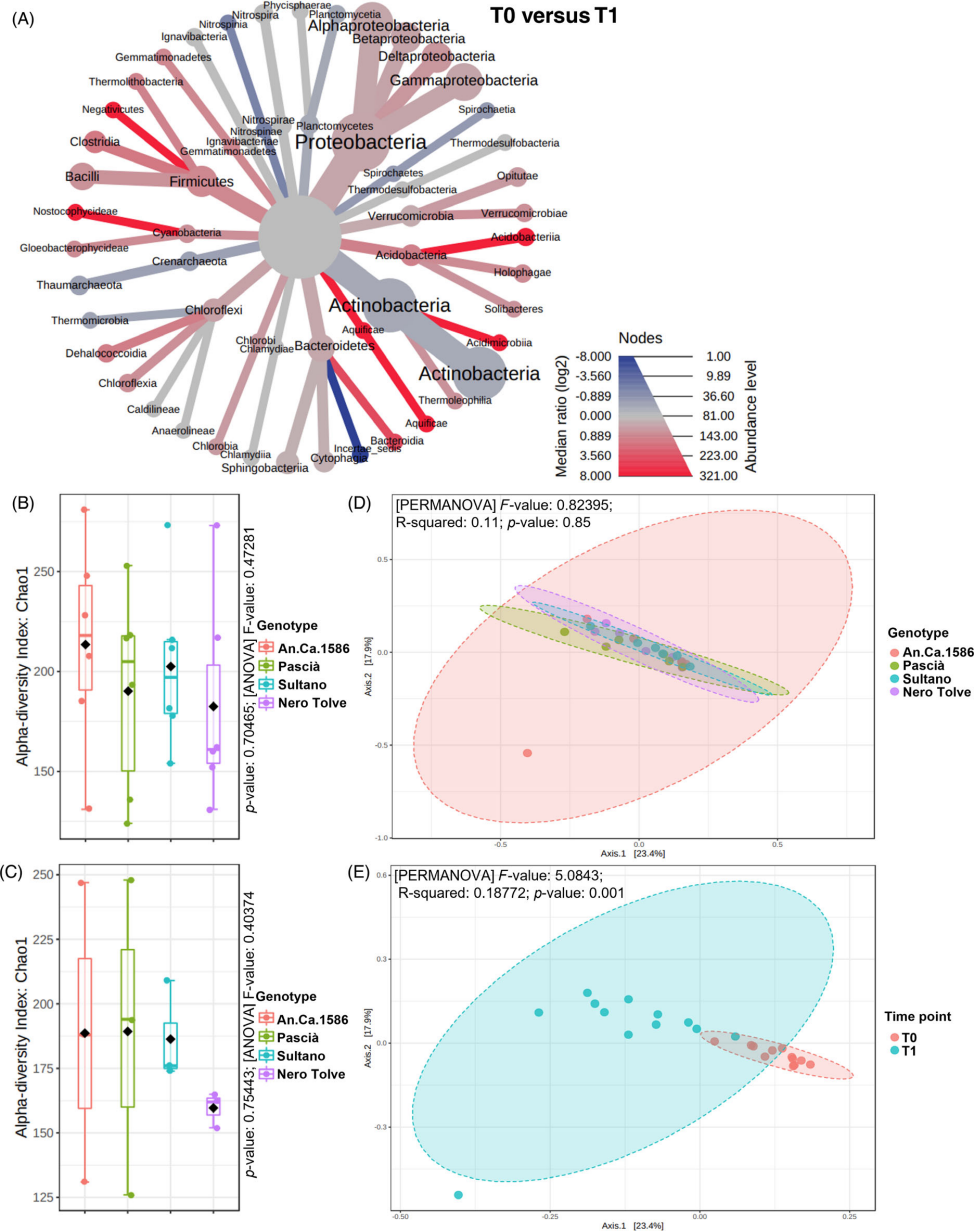
Heat tree, alpha and beta diversity of chickpea bulk soil microbiome. The heat tree (A) compares the abundances of the prokaryotic classes detected in chickpea bulk soil at T0 (July) versus T1 (August). The alpha diversity of the four chickpea genotypes (An.Ca.1586, Pascià, Sultano, Nero Tolve) was evaluated with the Chao1 index and significant differences were evaluated with ANOVA (B) and in August (T1, c). The beta diversity among the four chickpea genotypes (d) and among time points (E) was evaluated with the Jaccard index and significant differences were evaluated with PERMANOVA. The web‐based tool Microbiome Analyst was used (Dhariwal et al., 2017).

Analysis of the chickpea bulk soil bacterial community revealed that Actinobacteria (55%) and Proteobacteria (30%) were the dominant Phyla, followed by Bacteroidetes, Firmicutes, and Gemmatimonadetes (3%) (Figure S4). In T1 there was a slight increase in Actinobacteria (from 53% to 57%) and Crenarcheota (from 1% to 2%) compared to T0 and a slight decrease in Proteobacteria (from 30% to 29%) and in Firmicutes (from 4% to 2%) (Figure 3; Figure S4). The OTU table of soil samples is reported in Table S2.

There was no significant difference in the alpha diversity among the studied chickpea genotypes (*p*‐value: 0.70465; [ANOVA] *F*‐value: 0.47281; Figure 3), however, considering only the T1 data, the alpha diversity of the Nero Tolve genotype was strongly reduced compared to the others (especially An.Ca.1586 and Pascià) (Figure 3). Chickpea bulk soil beta diversity did not significantly differ among genotypes ([PERMANOVA] *F*‐value: 0.82395; R‐squared: 0.11; *p*‐value: 0.85; Figure 3), however, the beta diversity varied significantly when comparing the two‐time points ([PERMANOVA] *F*‐value: 5.0843; R‐squared: 0.18772; *p*‐value: 0.001; Figure 3), indicating that an alteration in species composition occurred over time. At T0, *Shinella fusca* was significantly more represented in the bulk soil of the An.Ca.1586 genotype (univariate analysis, *p*‐value 1.0251E‐4, FDR 0.033; Figure S5), compared to the others. At T1, the abundance of the genus *Mesorhizobium* was significantly higher in the Pascià genotype (univariate analysis, *p*‐value 6.5036E‐5; FDR 0.0060), compared to the other chickpea genotypes (Figure S5).

**FIGURE 4:**
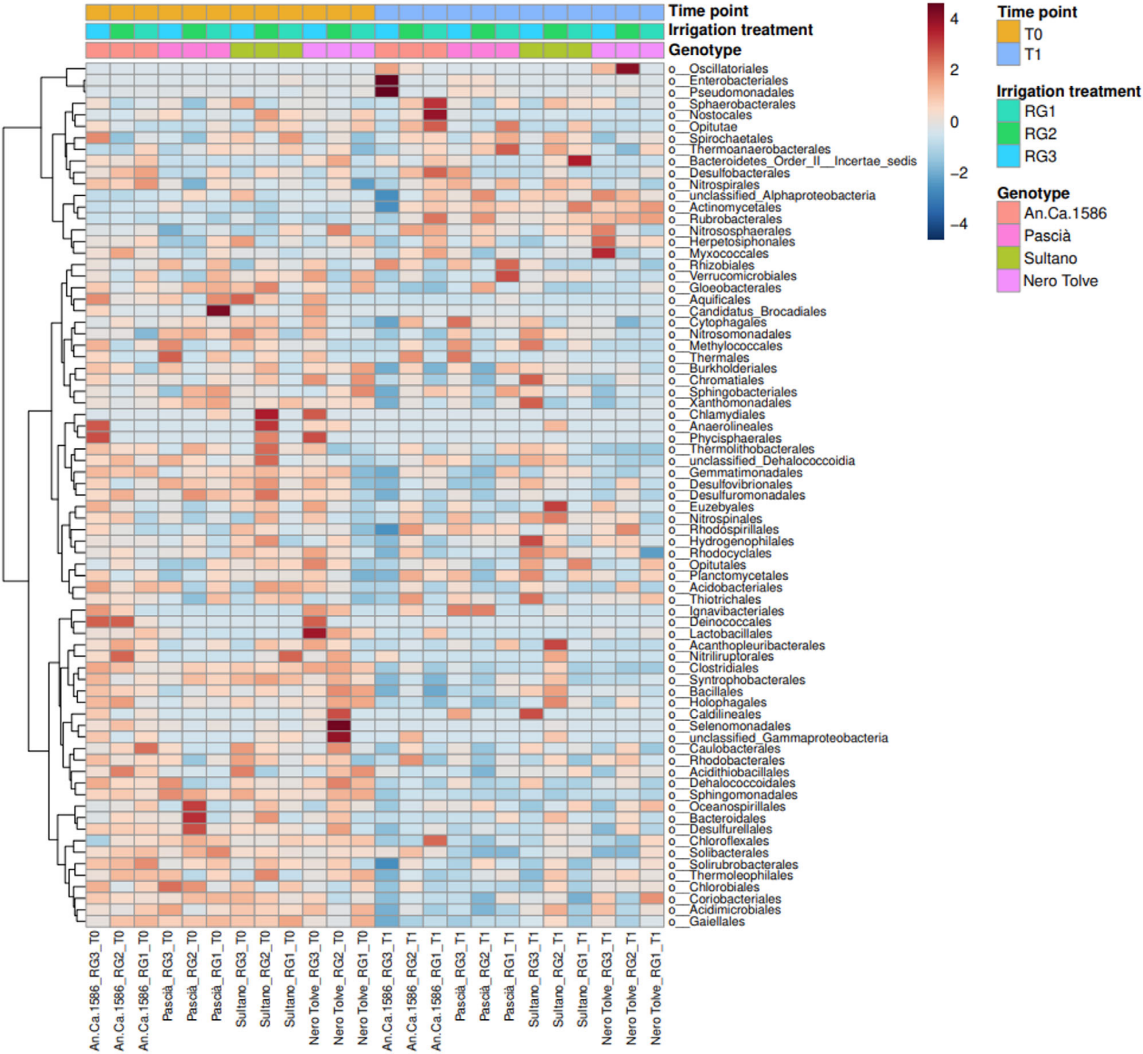
Heatmap of the genus abundance in chickpea bulk soil microbiome. The heatmap depicts the abundance (red high abundance, blue low abundance) of the prokaryotic genera in chickpea bulk soil microbiome. The heatmap is divided by sampling time point: July (T0) and August (T1). The irrigation treatments (RG) and the chickpea genotypes are also reported on the top of the heatmap. The T0 samples are constituted by a pool of four replicates, while at T1 only the replicate IV was sampled.

At the phylum level, a significant decrease in the abundance of the phyla Firmicutes (univariate analysis *p*‐value 5.3336E‐4; FDR 0.0086), Acidobacteria (univariate *p*‐value 9.1067E‐4; FDR 0.0086), Aquificae (univariate *p*‐value 0.0038182; FDR 0.024), Chlorobi (univariate *p*‐value 0.012036; FDR 0.047), and Gemmatimonadetes (univariate *p*‐value 0.012367; FDR 0.047) were observed at T1 (Figure S6). Significant differences were found also at Class level when comparing the two sampling time points. At T1 there was a significant reduction in the abundances of Deltaproteobacteria and Alphaproteobacteria (FDR 0.0090 and 0.017, respectively), Dehalococcoidia (FDR 0.017), Clostridia (FDR 0.0090), Gloeobacterophycideae (FDR 0.017), Solibacteres (FDR 0.017), Aquificae (FDR 0.023), and Chlorobia (FDR 0.023) (Figure S7). Comparing the two time points, there was a significant decrease in the second time point (T1) in the abundance of the genera *Sphingomonas* (Univariate *p*‐value 2.5326E‐7; FDR 2.5833E‐5*), Solirubrobacter* (univariate *p*‐value 2.4727E‐5; FDR 9.8423E‐4), *Lysobacter* (univariate *p*‐value 2.8948E‐5; FDR 9.8423E‐4), *Arenimonas* (univariate *p*‐value 2.6838E‐4; FDR 0.0063454), and *Ilumatobacter* (univariate *p*‐value 3.1105E‐4; FDR 0.0063454) (Figure S8). On the other hand, a significant increase in the abundance of the genus *Glycomyces* was found at T1 (FDR 0.046; Figure S8). This general decrease in the abundance of reads has been reported in the heatmap, which was generated by clustering results for each time point (Figure 4).

### Lentil bulk soil bacterial community

**FIGURE 5:**
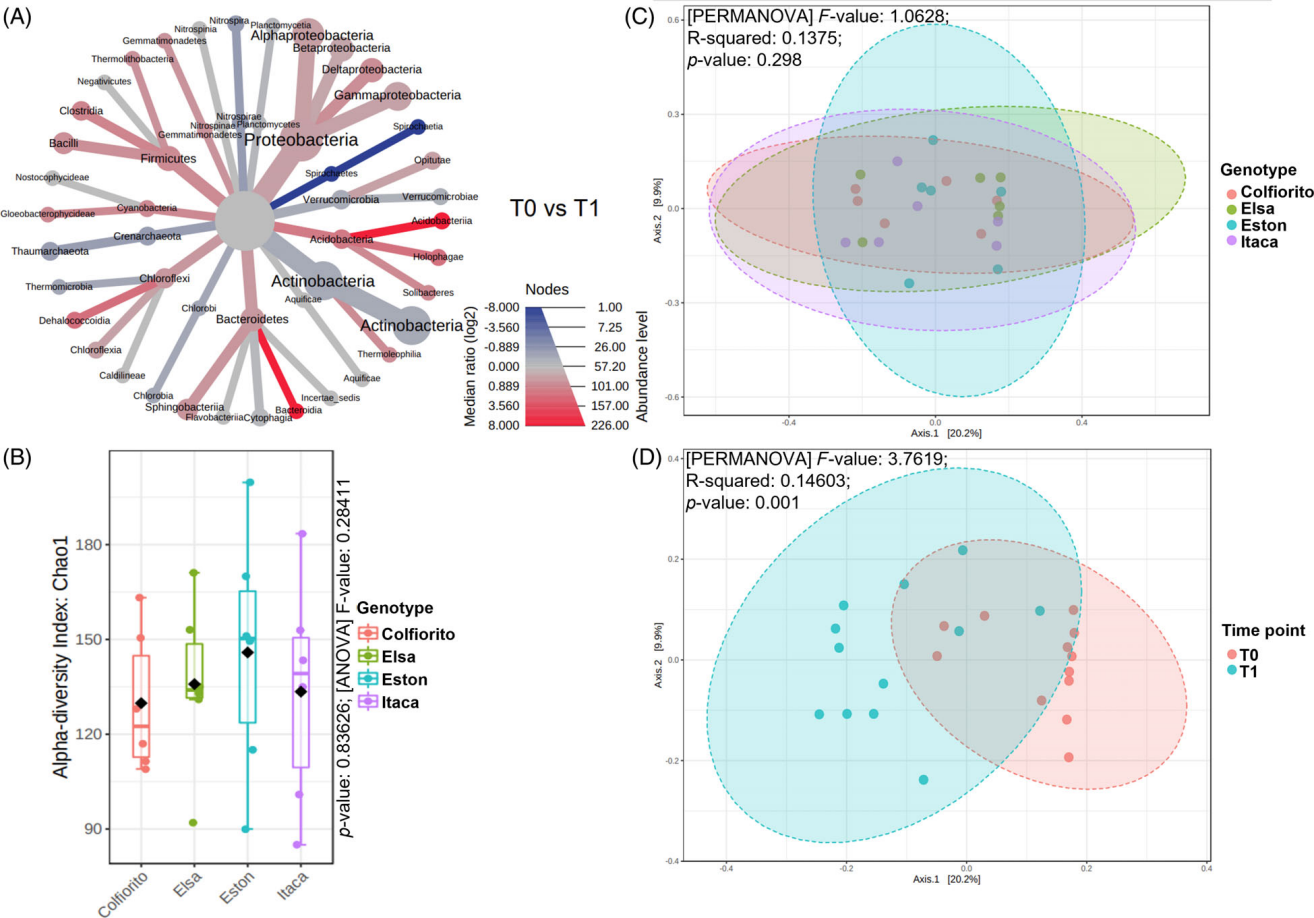
Heat tree, alpha and beta diversity of lentil bulk soil microbiome. The heat tree (A) compares the abundances of the prokaryotic classes present in lentil bulk soil at two time points: T0 (July) and T1 (August). The alpha diversity (B) of the four lentil genotypes (Colfiorito, Elsa, Eston, and Itaca) was evaluated with the Chao1 index and significant differences with ANOVA. The beta diversity between the four lentil genotypes (C) and between time points (D) was evaluated with the Jaccard index and significant differences were evaluated with PERMANOVA. The web‐based tool Microbiome Analyst was used (Dhariwal et al., 2017).

Analysis of the lentil bulk soil microbiome highlighted that Actinobacteria (49%) and Proteobacteria (29%) were the dominant phyla, followed by the less well‐represented Bacteroidetes (6%), Crenarchaeota (4%), Planctomycetes, and Firmicutes (3% and 3%) (Figure S9). At T1 there was a slight increase in *Actinobacteria* (from 47% to 52%) and Crenarcheota (from 4% to 5%) compared to T0, and slight decreases in *Proteobacteria* (from 31% to 28%), Bacterioidetes (from 7% to 5%), and Firmicutes (from 4% to 2%) (Figures 5 and S9). The OTU table of soil samples is reported in Table S3.

There was no significant difference in the alpha diversity among the studied lentil genotypes (*p*‐value: 0.83626; [ANOVA] *F*‐value: 0.28411), and a slight, but not statistically significant, reduction in the alpha diversity at T1 was displayed (*p*‐value: 0.35996; [t‐test] statistic: 0.93679; Figure 5).

Considering genotypes as a factor, lentil bulk soil beta diversity did not distinguish differences between genotypes (Figure 5). However, when comparing the two sampling time points a significant difference was observed ([PERMANOVA] *F*‐value: 3.7619; R‐squared: 0.14603; *p*‐value: 0.001; Figure 5), indicating that a shift in the bulk soil microbial community composition occurred during this time. At the class level, a significant reduction by T1 was registered for the Alphaproteobacteria family (*p*‐value 7.7919E‐4; FDR 0.025713) (Figure S10). At T1, there was a significant decrease in the abundance of the orders Sphingomonadales (FDR 2.5755E‐6), Gaiellales (FDR 1.3303E‐4), and Clostridiales (FDR 0.006791) (Figure S10). On the other hand, there was an increase in the abundance of the Rubrobacterales (FDR 0.0053701) (Figure S10). These changes occurring at T1 were partly reflected at family level. Indeed, there was a significant decrease in the abundance of Sphingomonadaceae (*p*‐value 3.7173E‐8, FDR 4.312E‐6), Gaiellaceae (*p*‐value 3.8319E‐6; FDR 2.2225E‐4), Geobacteraceae (*p*‐value 4.4418E‐4; FDR 0.012881), and Clostridiaceae (*p*‐value 0.0010575; FDR 0.024535) (Figure S11). Conversely, a significant increase in the Rubrobacteraceae family abundance was observed at T1 (*p*‐value 2.7014E‐4; FDR 0.010445) (Figure S11). At the genus level, *Sphingomonas* was less prevalent at T1 and was the only notable reported abundance shift (FDR 0.0031291) (Figure S11).

Looking at the T0 data, alpha diversity was significantly different among the studied lentil genotypes (*p*‐value: 0.99728; [ANOVA] *F*‐value: 0.015016) (Figure S12). Elsa and Colfiorito alpha diversity was reduced in comparison with Eston and Itaca genotypes. A similar trend was also observed at T1 (*p*‐value: 0.99675; [ANOVA] *F*‐value: 0.016914) (Figure S12). Regarding beta diversity, no significant difference among genotypes was found at both time points (Figure S12).

### Fungal functional annotation of the lentil root samples

Since many lentil parcels were suffering and showed disease symptoms (Figure S13) probably due to the presence of pathogens, an in‐depth analysis of the putative functions of lentil root‐associated microbiota at T1 was performed, considering both fungal and prokaryotic sequences (Tables S4, S5).

**FIGURE 6:**
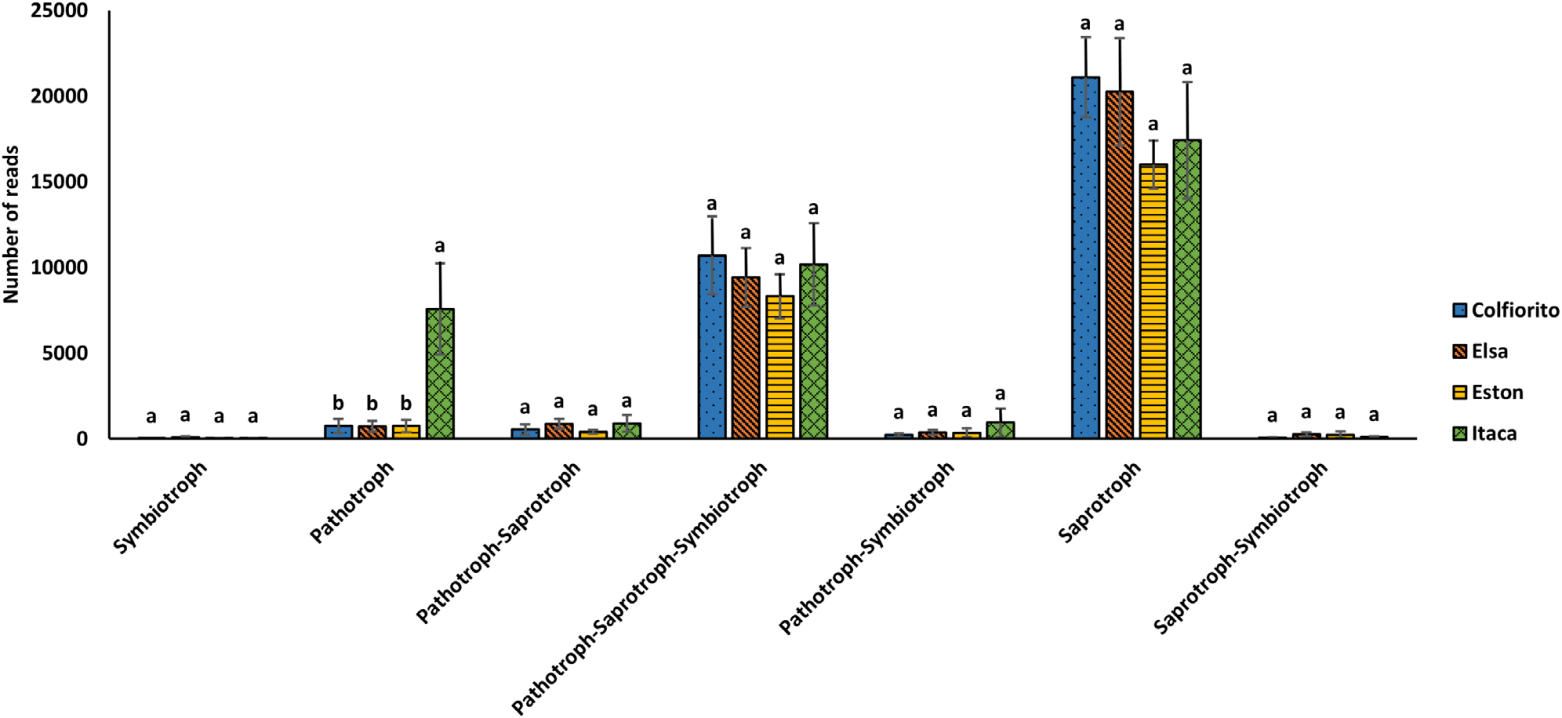
Fungal functional annotation in lentil roots. The histogram displays the fungal functional annotation obtained with the FUNGuild tool of lentil roots of diverse genotypes (Colfiorito, Elsa, Eston, and Itaca). Letters were assigned according to Kruskal–Wallis test and Dunn's post‐hoc test Bonferroni corrected.

The fungal functional annotation tool FUNGuild highlighted that saprotrophs were the most abundant inhabitants of all the lentil root samples (Figure 6), independently from the genotype. Pathotrophs‐Saprotrophs‐Symbiotrophs were also abundant in all lentil genotypes. Interestingly, pathotrophic fungi, including plant pathogens, were significantly more abundant in Itaca roots compared to the other genotypes (Kruskal–Wallis *p*‐value: 0.01043; Dunn's post‐hoc test Bonferroni corrected, Figure 6).

**FIGURE 7:**
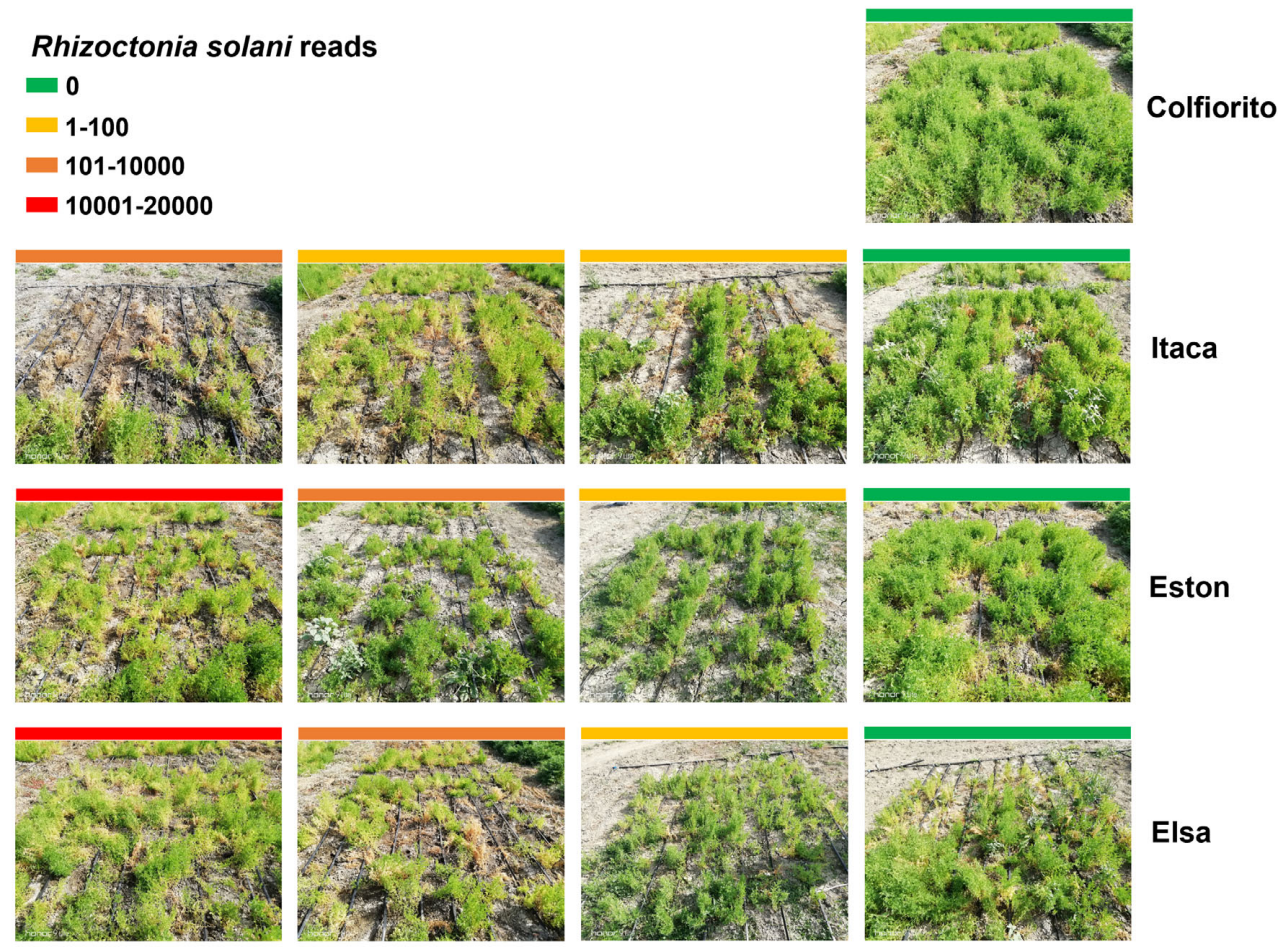
Visual comparison of lentil plant health with number of *Rhizoctonia solani* reads. The figure shows pictures of healthy and suffering lentil parcels in comparison with the number of *R. solani* reads found in each parcel. Each parcel measured 3 m × 3 m. Green indicates the absence of *R. solani* reads, yellow indicates a range of 1–100 reads, orange 101–10,000 reads, red 10,001–20,000 reads.

Notably, *Rhizoctonia solani* was detected in Itaca, Elsa, and Eston roots, while no *R. solani* reads were recorded in the Colfiorito genotype. In Figure 7, lentil plant health and a number of *R. solani* reads found in each sample were visually compared. Among fungal pathogens, *Macrophomina phaseolina* was present in all studied lentil genotypes, but only in one Eston sample. *Fusicolla septimanifiniscientiae* was identified in all lentil genotypes but only in one Colfiorito sample. *Fusarium* spp. were  abundant in roots of all lentil genotypes. For instance, *Fusarium oxysporum* was detected in all genotypes, with a maximum of 5195 reads in one Eston root sample (Eston IV RG2), *Fusarium falciforme* was very abundant in all lentil roots, showing up to 29,719 reads in one Elsa sample (Elsa I RG3), *Fusarium croci* reads were detected in all lentil genotypes, with a maximum of 2069 reads in a Colfiorito sample (Colfiorito IV RG3).

### Prokaryotic functional analysis in lentil root samples

**FIGURE 8:**
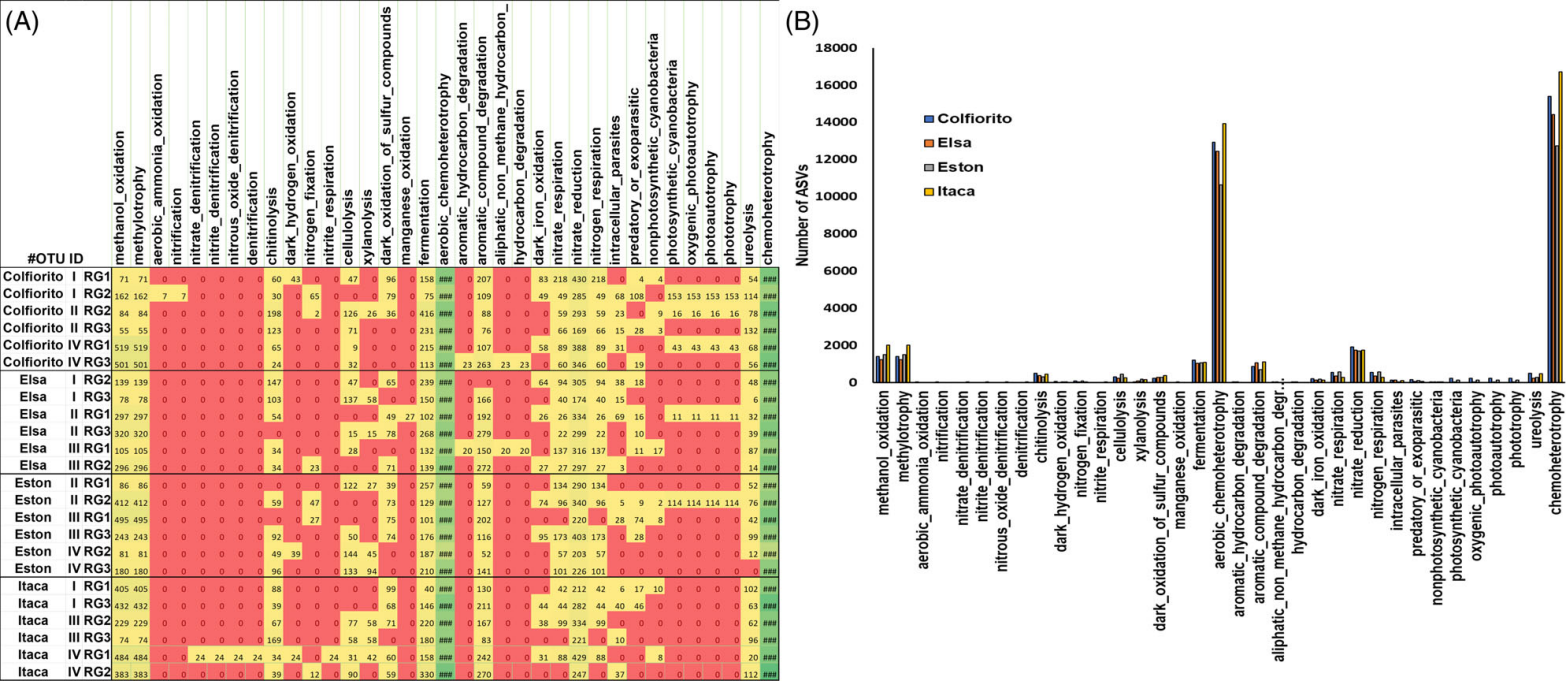
Prokaryotic functional annotation in lentil roots. The heatmap (A) and the histogram (B) display the prokaryotic functional annotation obtained with the FAPROTAX tool of lentil roots of diverse genotypes (Colfiorito, Elsa, Eston, and Itaca). In the heatmap colour scale, green colour corresponds to a high number of ASVs, while red corresponds to 0. The histogram (B) shows the number of ASVs corresponding to each FAPROTAX functional category of lentil genotypes (Colfiorito in blue, Elsa in orange, Eston in grey, and Itaca in yellow colour).

The prokaryotic functional annotation tool FAPROTAX revealed that among the lentil genotypes, no significant patterns in the prokaryotic ecological functions were observed (Figure 8). However, it was possible to observe that the most represented functional categories were those of chemoheterotrophy and aerobic chemoheterotrophy, followed by methanol oxidation, methylotrophy, and nitrate reduction (Figure 8). Other categories linked to the nitrogen cycle were detected, even if less well represented, such as nitrate, nitrogen respiration, and ureolysis. The categories related to fermentation and aromatic compound degradation were also observed, as well as categories related to the lysis of cellulose, xylan, and chitin.

### Analysis of common and specific prokaryotic families in lentil roots and bulk soil

**FIGURE 9:**
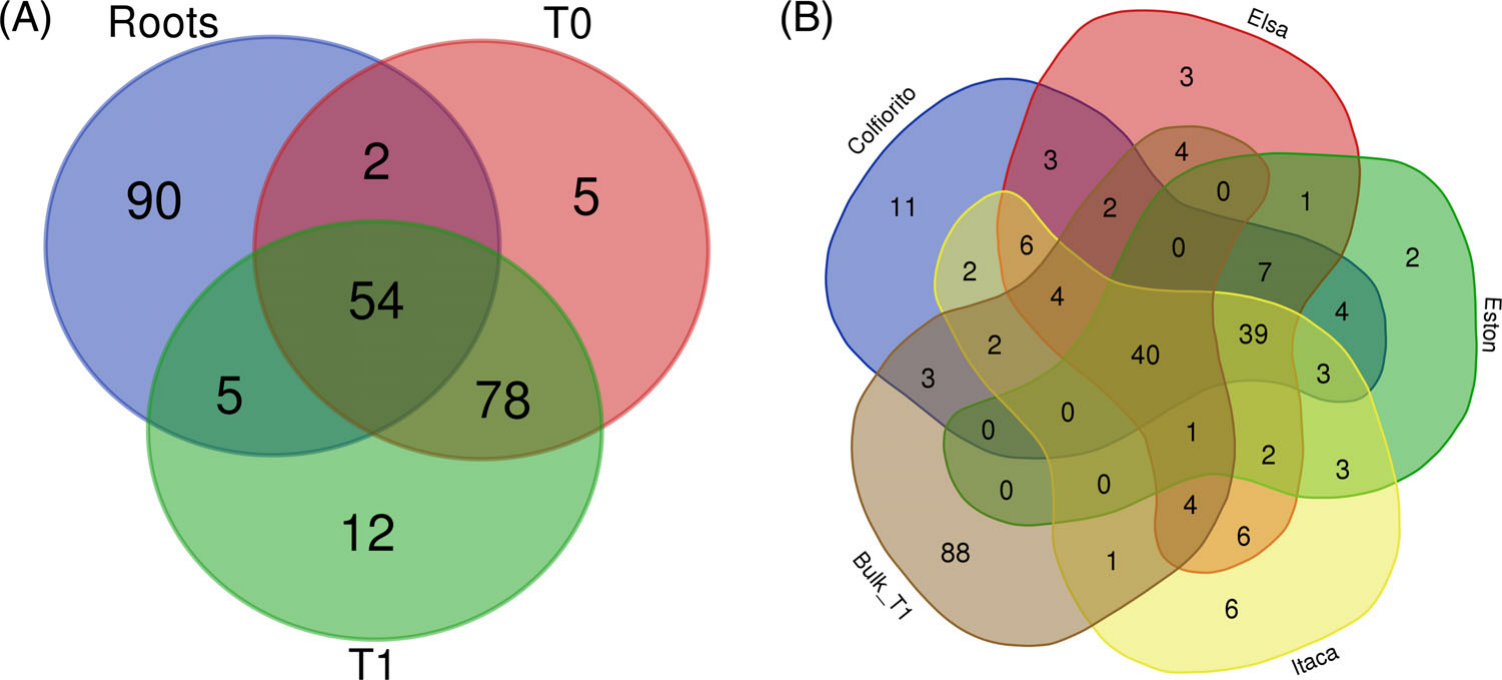
Venn diagrams of prokaryotic families of lentil roots and bulk soil. The Venn diagram (A) displays the common and specific prokaryotic families of lentil roots (August—T1) and bulk soil at different time points (July—T0, August—T1). The Venn diagram (B) shows the common and specific prokaryotic families of lentil bulk soil at T1 and roots from each lentil genotype (Colfiorito, Elsa, Eston, and Itaca) at T1. The Venn diagram was created with the online‐based tool https://bioinformatics.psb.ugent.be/webtools/Venn/.

A Venn diagram representing the common and specific prokaryotic families of the lentil roots and of the bulk soil at T0 and T1 was generated (Figure 9, Table S6). In total, in bulk soil 139 prokaryotic families were observed at T0 and 149 families were detected at T1, while 151 families were detected in lentil roots at T1. Among the latter, 90 families were exclusively found in the root compartment, for example, Bacteriovoracaceae, Cellvibrionaceae, Erwiniaceae, Rubrobacteriaceae, and Xanthobacteraceae. Two families were shared between roots and bulk soil at T0, that is, Glycomycetaceae and Phaselicystidaceae. Five families were shared between the roots and bulk soil at T1, that is, Alcaligenaceae, Alicyclobacillaceae, Planococcaceae, Polyangiaceae, and Saprospiraceae.

Five prokaryotic families were specifically associated to bulk soil at T0 (i.e., Desulfobacteraceae, Lachnospiraceae, Neisseriaceae, Rhabdochlamydiaceae, and Thermaceae), while 78 families were shared between bulk soil at T0 and T1 (e.g., Bradyrhizobiaceae, Cellulomonadaceae, Methylobacteriaceae, Phyllobacteriaceae, and Rubrobacteraceae). Instead, 12 prokaryotic families were specifically found in bulk soil at T1, such as *Aciditerrimonas*, Alteromonadaceae, Leuconostocaceae, Microthrixaceae, and Nitriliruptoraceae.

On the other hand, 54 prokaryotic families were common to all studied compartments (roots and bulk soil). Some examples are Bacillaceae, Burkholderiaceae, Chitinophagaceae, Hyphomicrobiaceae, Pseudomonadaceae, Rhizobiaceae, and Xanthomonadaceae.

An additional Venn diagram comparing the prokaryotic families present in root samples from each lentil genotype with the bulk soil samples at T1 highlighted the presence of 149 prokaryotic families in the bulk soil (T1), 126 families in Colfiorito samples, 122 families in Elsa samples, 102 families in Eston samples and 119 families in Itaca samples (Figure 9, Table S7). Eleven prokaryotic families were specific to Colfiorito root samples, including Phaselicystidaceae, and Bacteriovoracaceae. In addition, there were three families shared among Colfiorito and bulk soil T1 samples (i.e., Burkholderiaceae, Nitrososphaeraceae, and Hyphomonadaceae). Six prokaryotic families were specifically found in Itaca root samples, such as Alicyclobacillaceae and Vampirovibrionaceae. Three families were specific to the roots of Elsa, for example, Nitrosococcaceae, while four families were shared among Elsa and bulk soil T1 samples, for example, Polyangiaceae. On the other hand, only two prokaryotic families were found specifically in Eston roots, for example, Obscuribacteraceae. A core lentil microbiome was constituted by 40 families shared by all lentil genotypes and bulk soil, for example, Chitinophagaceae, Rhizobiaceae, Rubrobacteraceae, Bacillaceae, Xanthomonadaceae, and Pseudomonadaceae, and by 39 prokaryotic families specific to all lentil root genotypes but not present in bulk soil, including Azospirillaceae, Xanthobacteraceae, Devosiaceae, and Methylophilaceae.

## DISCUSSION

An increase in intense weather events, such as heavy rain and flooding, as well as rarefaction of precipitation events are affecting our lives in both direct and indirect ways (Blenkinsop et al., 2021; Fang et al., 2019; IPCC et al., 2019; Taherkhani et al., 2020; Ummenhofer & Meehl, 2017). The alteration of precipitation regimes affects soil water availability and ecosystem response, including plants and plant‐associated microorganisms (Trivedi et al., 2022; Wu et al., 2011; Xu et al., 2018). The initial aim of our study was to assess the impact of different irrigation treatments on the soil microbiota associated with different chickpea and lentil genotypes. However, the experiment was disturbed by a major erratic rain event that temporarily flooded the experimental field. Indeed, some parameters of the soil chemical data were affected by the time of sampling. Differences were detected in pH, Na content, and EC. Salinity and the EC of the soil decreased at the second sampling point, as previously observed during flooding in field experiments (de León‐Lorenzana et al., 2017). The pH value increased, and this outcome has been reported as being typical after flooding (Ding et al., 2019), confirming the effect of the rainfall event on the considered soil. Although we were not able to evaluate the effects of different irrigation treatments, we had the opportunity to observe how an unexpected climate event negatively impacted the growth season of important legume crops.

### Chickpea and lentil bulk soil bacterial community structure was influenced over time

Since they produced flowers and fruits earlier than the other chickpea genotypes, An.Ca.1586 and Pascià could be considered early varieties compared to Nero Tolve and Sultano. Model simulations predict a reduction in the crop growing season in African regions due to climate change and a generally negative effect in Europe, Southern Africa, and Australia (Ray et al., 2019). The need to develop new local adaptation strategies to face climate change effects on crop yields has been previously highlighted (Mourtzinis et al., 2015; Thornton et al., 2006). Therefore, the selection of chickpea varieties with early phenology, such as An.Ca.1586 and Pascià, might be a solution to attain the optimal time frame for the legume growing season. Regarding lentil plants, fruit production was observed exclusively in the Colfiorito and Elsa genotypes. During our experiment, many lentil plants, particularly belonging to Itaca genotype, showed shoot die‐back, suggesting, mainly due to the root features, that a fungal or a bacterial disease could be the cause.

The bulk soil prokaryotic community differed significantly when comparing the two crop species. A significantly higher alpha diversity was recorded for the chickpea genotypes compared with lentils. In addition, the chickpea and lentil genotypes clustered differently in the beta diversity analysis. However, it was possible to identify a chickpea and lentil bulk soil core microbiome mostly represented by Actinobacteria and Proteobacteria. These phyla are very common in agricultural soils (Zhao et al., 2019); Actinobacteria tend to be abundant when nitrogen availability in soil is high (Dai et al., 2018). In both chickpea and lentil bulk soil there was a slight increase in the relative abundance of Actinobacteria over time. In addition, the phylum Crenarchaeota doubled its abundance at T1 in chickpea bulk soil. Like Actinobacteria, Crenarchaeota were found to be favoured by nitrogen availability in soil (Cai et al., 2021), and facilitate soil nitrification (He et al., 2007; Leininger et al., 2006). The soil of the experimental field contained an average amount of nitrogen, which may have supported the presence of both phyla.

A decrease in the relative abundances of Proteobacteria and Bacterioidetes was observed in chickpea and lentil bulk soil over time. Different life strategies of these phyla may explain this shift in bacterial community composition. Bacteroidetes and Proteobacteria are generally considered copiotrophic microorganisms, thriving when nutrient availability is high (Chen et al., 2015; Eilers et al., 2010; Fierer et al., 2007; Nemergut et al., 2010). Nutrients are generally known to be more abundant in the rhizosphere, rather than in bulk soil. Therefore, the growth of these phyla is favoured by the proximity of the plant roots (Mimmo et al., 2018; Uren, 2000; van Hees et al., 2005), as also highlighted by the abundance of several bacterial families belonging to these phyla in lentil roots.

In the experimental chickpea field, two members of the Rhizobiaceae family, that is, *Shinella fusca* and *Mesorhizobium*, were found to be significantly more abundant in the bulk soil of two genotypes (An.Ca.1586 and Pascià) compared to the others. *Shinella* and *Mesorhizobium* are known to establish a symbiosis with legumes (Muleta et al., 2022; Wang, 2019). Particularly, *Mesorhizobium* has been reported to specifically nodulate with chickpea plants (Muleta et al., 2022; Yadav et al., 2013, 2021). When rhizobia form effective nodules in the plant roots, the bacteria fix atmospheric nitrogen in the form of ‘ready‐to‐use’ ammonia. This symbiosis is particularly important in nutrient‐deficient soils to support plant growth (Mozumder et al., 2003). Ogola et al. (2021) showed that the inoculation of chickpea plants with rhizobia could significantly increase grain yield. The raise in productivity in terms of fruits and flowers of An.Ca.1586 and Pascià genotypes might be explained by the presence of these taxa. The presence of members of the Rhizobiaceae family in the soils related to these genotypes may have increased soil nutrient availability, therefore increasing plant productivity. There were no significant differences in the alpha diversity of the bulk soil of the studied chickpea and lentil genotypes. However, for both chickpea and lentil bulk soils, a difference in beta diversity was observed over time.

A significant reduction in the relative abundance of the phylum Firmicutes at T1 for both the chickpea and lentil bulk soil microbiome was observed. This phylum is very common in soil, especially in plant rhizosphere (Kumar et al., 2012). Members of the phylum Firmicutes display many beneficial traits to promote plant growth in the presence of abiotic and biotic stress (Hashmi et al., 2020). It is widely demonstrated that plants can recruit beneficial microbes in the rhizosphere through root exudates, ‘crying for help’ during their growth as sessile organisms (Girsowicz et al., 2019; Li et al., 2014; Shi et al., 2015). An intriguing hypothesis for the halving in the relative abundance of Firmicutes at T1 could be that chickpea and lentil plants are attracting beneficial bacteria towards their roots, causing a decrease in their abundance in the bulk soil. A similar explanation may be given to the decrease in the relative abundance of the phylum Acidobacteria in chickpea bulk soil. A recent study identified Acidobacteria as an important bacterial taxon in soil with a role in the decomposition of soil organic matter and denitrification, enhancing nutrient availability in soil (Banerjee et al., 2018). In addition, in chickpea bulk soil there was a significant decrease over time in the relative abundances of other beneficial bacterial genera such as *Arenimonas*, *Lysobacter*, and *Sphingomonas*. Similarly, also in lentil bulk soil, there was a decrease in the relative abundance of *Sphingomonads* over time (order, family, and genus level), they are ubiquitous in the environment and have wide metabolic capabilities, being able to degrade recalcitrant organic carbon sources (Fredrickson et al., 1999; White et al., 1996). *Lysobacter* spp. is a promising bacterial biocontrol agent, due to its vast capability to produce secondary metabolites and lytic enzymes (Vasilyeva et al., 2014; Xie et al., 2012; Zhang et al., 2011), and its presence has been associated with disease suppressiveness in soils (Gómez Expósito et al., 2015). The genus *Arenimonas* is considered beneficial for plant growth and can enhance soil recovery due to its ability to solidify or mineralize heavy metals (Chen, Shi, & Wang, 2012; Radziemska et al., 2021; Stec et al., 2000).

The abundance of the genus *Solirubrobacter* was also significantly reduced in the chickpea bulk soil over time. Members of this genus are reported to be sensitive to abiotic stress since they are incapable of forming spores (Nunes et al., 2018; Singleton et al., 2003). Their low resistance to high temperatures might explain the decrease in the relative abundance of this genus at T1. Indeed, at the end of July, maximum air temperature was relatively high (with a peak of 41.07°C reached on 29 July 2021).

On the other hand, *Glycomyces* was the only genus whose relative abundance increased in chickpea bulk soil over time. *Glycomyces* is a genus of the Actinobacteria commonly found in the rhizosphere or as endophytic bacteria, and it has also been isolated from extreme environments (Guan et al., 2011; Mu et al., 2018; Qin et al., 2008; Sorokin et al., 2021; Zhang et al., 2014).

### Phytopathogenic fungi enriched lentil root samples

During the experiment, several lentil plants (especially belonging to the Itaca genotype) were showing chlorotic leaves and necrotic roots, and many plants in some parcels died. It could be hypothesized that these symptoms could be due to a fungal or bacterial disease. Therefore, a metabarcoding analysis on fungal and bacterial DNA isolated from lentil roots was performed. The fungal functional annotation analysis revealed that the experimental lentil field was affected by fungal pathogens, but the Itaca genotype showed substantially more reads corresponding to them than the rest of the field. In particular, the presence of *R. solani* reads was observed only in Itaca, Elsa, and Eston samples and a visual inverse correlation between *R. solani* reads and plant health was established. The broad‐spectrum plant pathogen *R. solani* has already been documented as a destructive pathogen causing lentil root‐rot and damping‐off (Chang et al., 2008; Channa et al., 1995; Duarte et al., 2018) and was reported to cause lentil root and collar rot in Italy in 2002 by Tosi et al. (2002). Soil humidity is pivotal for the survival of this fungus, even if soil moisture may not influence disease severity (Feng et al., 2017; Paula & Hau, 2007). Since different lentil genotypes were cultivated in randomized parcels, it is unlikely that *R. solani* infected three out of four lentil genotypes due to their spatial disposition in the field. One possible explanation could be that Itaca, Elsa, and Eston might be particularly susceptible genotypes to *R. solani*, while Colfiorito might display some tolerance to the disease due to genetic traits.

*Rhizoctonia solani* was not the only phytopathogenic fungus inhabiting the experimental field. Not surprisingly, there was a high abundance of *Fusarium* spp. reads. The Ascomycete *Fusarium* spp. is widely distributed in soil, as it can survive for many years with a saprotrophic behaviour (Goncharov et al., 2020; Stack & McMullen, 1985). *Fusarium oxysporum* has been reported to cause fusarium vascular wilt in lentils (Bayaa & Erskine, 1998). The optimal environmental conditions for this pathogen (i.e., warm temperatures and low humidity) may lead to the complete loss of the crop yield (Chen et al., 2011). During summer 2021, air humidity was relatively high (ranging from a minimum of 36.58% registered at 01/07/2021, to a maximum of 81.91% obtained at 19/07/2021), hence, it is unlikely that the optimal condition for *F. oxisporum* infection occurred. However, the pathogen was strongly present in the soils, representing a threat for the crops in the experimental field. Other detected *Fusarium* species in lentil root samples were *F. croci* and *F. falciforme*. To our knowledge, these two *Fusarium* species have never been reported on lentils. However, their presence suggests that the strategy of crop rotation might not be enough to avoid the excessive proliferation of *Fusarium*‐related pathogens in soil.

Interestingly, reads of the fungal pathogen *Macrophomina phaseolina* were detected as associated to the roots of each lentil genotype. However, this pathogen was present with a very low abundance in Elsa samples, suggesting that some form of tolerance to this pathogen might be present. A possible explanation might be that Elsa root exudates differ from the other lentil genotypes in their composition, hindering the development of the pathogen in the environment surrounding the roots. *Macrophomina* genus is included in the Botryosphaeriaceae family, and *M*. *phaseolina* is a broad‐spectrum soil‐borne fungal pathogen that attacks different legume plants, including lentils, as it can survive for many years in the environment in the form of microsclerotia (Ali & Dennis, 1992; Tonin et al., 2013; Ullah et al., 2019). Optimal conditions for *M*. *phaseolina* growth and survival include low soil moisture and a temperature of 35°C–40°C has been reported to trigger microsclerotia virulence (Dhingra & Sinclair, 1974; Olaya & Abawi, 1996). Therefore, in a climate change scenario in which reduced rainfall and global rises in temperature are predicted, it is likely that this pathogen will thrive and spread, adding a new threat to Mediterranean agriculture (Pandey & Basandrai, 2020; Pour et al., 2020).

### Chemoheterotrophy and aerobic chemoheterotrophy were the bacterial core functional groups more represented in lentil root samples

The FAPROTAX analysis showed that the lentil roots were populated by a bacterial core functional group of chemoheterotrophy, indicating that most lentil root‐associated bacteria can obtain carbon and energy from the oxidation of organic compounds available in the environment (Zhang et al., 2018). The functional category of nitrate reduction was highly represented in lentil roots. Soil microorganisms mainly mediate soil N cycle processes, and nitrate reduction may affect the production of greenhouse gases (e.g., N_2_O) (Johnson et al., 2005; Yoon et al., 2015). Moreover, nitrate reduction is an anaerobic process, and the high activity of this microbial process seems to be associated with the temporary flooding of the experimental field (Cabello et al., 2009). The anoxic condition of the experimental field was further reflected in the high activity of methanol oxidation and methylotrophy functions in the lentil root prokaryotic community. Methanotrophs and methylotrophic prokaryotes are typically abundant in paddy fields and wetlands (Iguchi et al., 2012; Kirschke et al., 2013; Lee et al., 2014). Methanotrophs consume methane as an energy source, preventing its release into the atmosphere and reducing methane gas emissions (Davamani et al., 2020). On the other hand, methylotrophs use methane‐derived carbon (e.g., methanol) as an energy source, and they are reported to increase the methane oxidation rate (Krause et al., 2017). Another well‐represented function in the lentil roots was fermentation. This is a typical anoxic process that has been observed in paddy fields (Ji et al., 2018). Although less well represented, functions related to cellulolysis, xylanolysis, chitinolysis, and intracellular parasitism were present in the lentil root‐associated prokaryotic community. Members of the bacterial families Xanthomonadaceae, Firmicutes, Pseudomonadaceae, and Bdellovibrionaceae may partly explain the occurrence of these ecological functions (Afoshin et al., 2020; Asmani et al., 2020; Dhivahar et al., 2020; Sockett, 2009).

### Nitrogen‐fixing bacteria constitute the lentil core prokaryotes, and each lentil genotype was characterized by a ‘unique’ prokaryotic community

The used metabarcoding approach allowed us to identify 54 families that constitute the prokaryotic community shared by all lentil root and bulk soil samples, similar to a previous study of *L*. *culinaris* root‐associated microbiome (Pramanik et al., 2020). More specifically, we identified a ‘core’ set of 40 families detected in all lentil genotypes and bulk soil at T1, suggesting that lentil roots successfully recruited these bacterial taxa from bulk soil, hosting them in the root niche. *Rhizobia*, the nitrogen fixers ‘par excellence’, belongs to the Rhizobiaceae family, detected in both lentil root and bulk soil samples at T1. Recent studies reported that members of the Rhizobiaceae family are common in bulk soil and some species are recruited and selectively favoured by plant root exudates to colonize roots and establish a symbiosis (Miranda‐Sánchez et al., 2016; Regus et al., 2014). In our study, the Rhizobiaceae family was part of the lentil core prokaryotic community since all genotypes recruited this family in the root compartment. Bacillaceae, Pseudomonadaceae, and Xanthomonadaceae represent other families in the core prokaryotic community. The Bacillaceae family is ubiquitous in nature and includes resilient species able to survive in extreme environments thanks to their ability to form endospores (Rooney et al., 2009; Setlow, 2006). However, several taxa of Bacillaceae family survive as saprotrophs in soil, playing a relevant role in carbon, nitrogen, sulfur, and phosphorous cycles (Siala et al., 1974; Soares et al., 2012). Members of the Bacillaceae family are diverse and include not only human and animal pathogens, but also insect pathogens (e.g., *Bacillus thuringiensis*), allowing their use for pest management practices (Chattopadhyay et al., 2004; Ivanova et al., 2003). Moreover, several *Bacillus* species can be used as biofertilizers, as they can promote plant growth, and as biopesticides, inhibiting the growth of fungal pathogens (Choudhary & Johri, 2009; Hernandez et al., 2009; Ongena & Jacques, 2008; Sharma et al., 2012). Similarly, members of the Pseudomonadaceae and Xanthomonadaceae families are diverse in their ecological roles. They include human and animal pathogens (Mahar et al., 2010; Osawa et al., 2018), phytopathogenic species (Arnold & Preston, 2019; Van Sluys et al., 2003), plant‐beneficial species and strains with disease‐suppression capacity (Puopolo et al., 2014; Zhang et al., 2020).

In the detected core prokaryotic community, the Actinobacteria Rubrobacteraceae family encompasses bacteria able to tolerate periods of water scarcity through the accumulation of osmoprotectant metabolites (Meier et al., 2021). As lentil in Italy is usually a rainfed crop, it could be hypothesized that the Rubrobacteraceae may play an important role in the lentil rhizosphere by protecting the plant against periods of water shortage. The Chitinophagaceae family constitutes another interesting example in the lentil core prokaryotic community. This family is commonly found in soil and can degrade chitin (Sangkhobol & Skerman, 1981). This feature may be of interest for biocontrol since chitin is the major constituent of the fungal cell wall (Lenardon et al., 2010). On the other hand, various prokaryotic families were specifically found in the root compartment in all genotypes, but not in the bulk soil. Several of these families are characterized by the ability to perform biological nitrogen fixation. For instance, the Azospirillaceae family includes important plant growth‐promoting rhizobacteria (PGPR) genera that can colonize plant roots and have positive effects on their hosts by fixing nitrogen and producing plant hormones such as auxins (Fibach‐Paldi et al., 2012; Okon et al., 1983; Spaepen et al., 2014). In addition, Xanthobacteraceae was among the prokaryotic families exclusively identified in the root compartment of all lentil genotypes. The Xanthobacteraceae family includes several genera able to fix nitrogen and promote plant growth while protecting the plants from abiotic stress (Abd El‐Azeem et al., 2012; Dal Cortivo et al., 2017). Interestingly, also the Devosiaceae family was exclusively detected in the root compartment. This family is included in the α‐proteobacteria within the order Rhizobiales. Although, to our knowledge, this family has never been reported in lentils, some *Devosia* species have been reported to nodulate legume plants (Bautista et al., 2010; Negi et al., 2022; Rivas et al., 2002, 2003).

The Methylophilaceae family is characterized by obligate or restricted facultative methylotrophs capable of growth on carbon compounds such as methanol, methylamines, and dichloromethane (Doronina et al., 2014). These bacteria stimulate plant growth and development due to the production of bioactive substances such as phytohormones and vitamins, while plants simultaneously act as an important source of methanol for these bacteria (Agafonova et al., 2013; Doronina et al., 2002; Rani et al., 2021; Siddikee et al., 2010; Yurimoto et al., 2021).

In addition to the identification of a core prokaryotic community shared by all lentil genotypes, it was possible to distinguish four distinct unique microbial communities characterizing each different lentil genotype. Colfiorito was the genotype characterized by the highest number of unique prokaryotic families (11) compared to the other lentil genotypes. Colfiorito is an Italian lentil landrace, that is, a traditional local variety, characterized by a high level of genetic diversity (Sonnante & Pignone, 2007). Colfiorito genetic local adaptation might explain its better performance and microbial recruiting efficiency compared to the other commercial genotypes. Notably, two families well known to play a crucial role in the nitrogen cycle in soil were found to be exclusively associated to Colfiorito roots and bulk soil at T1, that is, Burkholderiaceae and Nitrososphaeraceae (Kerou & Schleper, 2016). Several *Burkholderia* species can fix nitrogen in soil and promote plant growth (Caballero‐Mellado et al., 2004; Santos et al., 2001). Some *Burkholderia* species have been reported to establish symbiosis with legume roots, fixing nitrogen inside root nodules (Chen et al., 2003). Part of the Colfiorito‐specific prokaryotic community, the Bacteriovoracaceae family, includes Bdellovibrio‐and‐like‐organisms that are water and soil‐inhabitant motile predatory bacteria (Davidov & Jurkevitch, 2004; Jurkevitch et al., 2000). Despite the ecology of these bacteria being poorly understood, it has been reported that they can lyse not only Gram‐negative bacteria but can also degrade and use as a nutrient source the biofilm produced by Gram‐positive bacteria (Chen, Young, et al., 2012; Im et al., 2018). Similarly, the Phaselicystidaceae family appears to have a bacteriolytic predatory behaviour (Garcia & Müller, 2014a), and it is thought to be a reservoir of new secondary metabolites and bioactive compounds for its close phylogenetic relatedness with the Polyangiaceae (Garcia & Müller, 2014b). Conversely, Colfiorito roots lacked of some bacterial families that were detected in the roots of the commercial genotypes. For instance, the Erwiniaceae family includes many pathogenic bacteria for plants, such as *Erwinia amylovora* (Zhao et al., 2009). Nevertheless, some members of this prokaryotic family are promising plant growth promoters (Saldierna Guzmán et al., 2021). Either the unique presence of specific families in the Colfiorito lentil root samples or the selective absence of other families may explain the enhanced tolerance of this genotype to the fungal pathogen *R. solani*, compared to the other ones. The specific prokaryotic community associated with the Itaca genotype included Alicyclobacillaceae and Vampirovibrionaceae. The latter is a predatory cyanobacteria family (Baer & Williams, 2015). Instead, Alicyclobacillaceae typically includes thermophilic and acidophilic bacteria that are reported to be mostly chemoorganotrophic, and a few strains can reduce nitrate to nitrite (Stackebrandt, 2014).

Elsa lentil root samples were characterized by the Nitrosococcaceae family. These are ammonia oxidizers usually associated with saline aquatic habitats; however, they have also been reported in soils (Pan et al., 2018; Sun et al., 2022). The Eston unique prokaryotic community was the most reduced in terms of the number of prokaryotic families in comparison with the other genotypes (showing just two specific families) and included the Obscuribacteraceae family, phylogenetically related to Cyanobacteria. This family is still poorly studied, but some species can metabolize polyphosphate (Soo et al., 2014).

The landraces inferior yields, pest and disease resistance, and postharvest shelf life in comparison with modern varieties are the reasons leading to a general decrease in use of locally adapted varieties in the cultivation of many horticultural crops (van de Wouw et al., 2010). However, Colfiorito landrace showed a higher fruit production, a potentially improved tolerance to *R. solani*, and a better microbial recruiting efficiency compared to the modern commercial lentil genotypes.

## CONCLUSIONS

Overall, our study highlighted the impact of an erratic rainfall event on the growth and productivity of important leguminous crops by affecting their bacterial community structure in a relatively short period of time, that is, less than 1 month. The presence of *Rhizobia* in the soil of two chickpea genotypes may have increased the availability of nutrients in soil, and the consequent productivity of these genotypes in terms of flowers and fruits. We described a core of prokaryotic families common to all lentil genotypes that included several taxa with nitrogen‐fixing ability, as well as a genotype‐specific prokaryotic community. A high number of bacterial taxa mostly associated to beneficial traits was exclusively detected in the Colfiorito genotype. This may be explained by the higher microbial recruiting efficiency of this locally adapted landrace compared to commercial genotypes. With the perspective of resilient and sustainable agriculture, the use of traditional lentil landraces and their specific microbial community may mitigate the negative effects on crop productivity due to climate change.

## AUTHOR CONTRIBUTIONS

**Francesca Brescia:** Data curation (equal); formal analysis (equal); investigation (equal); methodology (equal); writing – original draft (equal); writing – review and editing (equal). **Fabiano Sillo:** Data curation (equal); formal analysis (equal); investigation (equal); methodology (equal); writing – original draft (equal); writing – review and editing (equal). **Elisabetta Franchi:** Investigation (equal); methodology (equal); writing – review and editing (supporting). **Ilaria Pietrini:** Data curation (equal); formal analysis (equal); investigation (equal); writing – review and editing (supporting). **Vincenzo Montesano:** Investigation (supporting); writing – review and editing (supporting). **Giovanni Marino:** Formal analysis (equal); investigation (equal); writing – review and editing (supporting). **Matthew Haworth:** Formal analysis (equal); investigation (equal); writing – review and editing (supporting). **Elisa Zampieri:** Investigation (equal); writing – review and editing (supporting). **Danilo Fusini:** Formal analysis (equal); investigation (equal); writing – review and editing (supporting). **Martino Schillaci:** Investigation (supporting); writing – review and editing (supporting). **Roberto Papa:** Resources (lead); writing – review and editing (supporting). **Chiara Santamarina:** Resources (supporting); writing – review and editing (supporting). **Federico Vita:** Investigation (supporting); writing – review and editing (supporting). **Walter Chitarra:** Methodology (equal); writing – review and editing (supporting). **Luca Nerva:** Methodology (equal); writing – review and editing (supporting). **Giannantonio Petruzzelli:** Investigation (equal); writing – review and editing (supporting). **Carmelo Mennone:** Methodology (supporting); writing – review and editing (supporting). **Mauro Centritto:** Conceptualization (supporting); funding acquisition (supporting); methodology (equal); writing – review and editing (supporting). **Raffaella Balestrini:** Conceptualization (lead); funding acquisition (lead); investigation (equal); methodology (equal); writing – review and editing (lead).

## CONFLICT OF INTEREST STATEMENT

The authors have no conflict of interest to declare.

## Supporting information


**Table S1.** Climatic data are reported for the period from 01 June 2021 to 30 September 2021. The sowing date was at 04 June 2021. For each day (DD/MM/YYYY), the rain (mm), the average direct solar radiation (Wm^−2^), the minimum, maximum, and average air temperature (°C), the average relative humidity (%), the wind speed (m s^−1^) and the reference evapotranspiration (Eto, mm) calculated with FAO Penman–Monteith method and Hargreaves method are reported.

**Table S2.** The operational taxonomic unit (OTU) of chickpea bulk soil at T0 and T1 is reported.
**Table S3.** The operational taxonomic unit (OTU) table of lentil bulk soil samples is reported.
**Table S4.** The amplicon sequence variant (ASV) table of lentil root samples referring to fungal sequences is reported.
**Table S5.** The amplicon sequence variant (ASV) table of lentil root samples referring to bacterial sequences is reported.

**Table S6.** List of prokaryotic families specific and shared among lentil roots, bulk soil at T0 (July), and bulk soil at T1 (August). The ‘Compartment(s)’ column indicates the specific or shared sample type, the ‘Number of elements’ column indicates the number of prokaryotic families found in each compartment, and in the ‘Families’ column the prokaryotic families are listed. The Venn diagram table was obtained through the https://bioinformatics.psb.ugent.be/ web‐based tool.

**Table S7.** List of prokaryotic families specific and shared among lentil root samples, bulk soil at T1 (August). The ‘Compartment(s)’ column indicates the specific or shared sample type, the ‘Number of elements’ column indicates the number of prokaryotic families found in each compartment, and in the ‘Families’ column the prokaryotic families are listed. The Venn diagram table was obtained through the https://bioinformatics.psb.ugent.be/ web‐based tool.

**Figure S1.** Meteorological data. In the graph (a) the mm of rain for each day of the experimental period (from 04 June 2021 to 10 August 2021) are reported. In the graph (b) the minimum, maximum, and average air temperature (°C), as well as the average relative humidity (%), are displayed for each day of the experimental period.
**Figure S2.** Scatterplots of chickpea agronomic and eco‐physiological measures. Chickpea genotype (An.Ca.1586, Nero Tolve, Pascià, Sultano) heights (cm) were measured in July and August 2021 (T0 and T1, respectively) (a)–(d). Chickpea genotype stomatal conductance (gsw) was measured at T0 and T1 (e)–(h). Irrigation treatments (RG) are reported in different colours as 100% water, not stressed (RG1), 50% water (RG2), and 25% water (RG3).
**Figure S3.** Scatterplots of lentil height. Lentil genotype (a: Colfiorito, b: Elsa, c: Eston, d: Itaca) heights (cm) were measured in July and August 2021 (T0 and T1, respectively). Irrigation treatments (RG) are reported in different colours as 100% water, not stressed (RG1), 50% water (RG2), and 25% water (RG3).
**Figure S4.** Heat tree and pie charts of chickpea bulk soil microbiome. The heat tree of the prokaryotic phyla present in chickpea bulk soil (a) and the pie charts (b)–(d) were generated with the web‐based tool Microbiome Analyst (Dhariwal et al., 2017). The heat tree (a) depicts the hierarchical structure of phyla and the relative abundance of chickpea bulk soil microbial communities. The colour gradient and the size of node, edge, and label are based on the log_2_ ratio of median abundance. The pie chart (b) shows the taxonomic abundance of chickpea bulk soil in both time points (T0 and T1). The pie charts (c), (d) show the taxonomic abundance of chickpea bulk soil at T0 (July, c) and at T1 (August, d).
**Figure S5.** Univariate analysis at feature level of chickpea bulk soil microbiome in July. The univariate analysis of the four chickpea genotypes (An.Ca.1586, Pascià, Sultano, Nero Tolve) was evaluated for the first time point in July (T0, a) and for the second time point in August (T1, b) with the web‐based tool Microbiome Analyst (Dhariwal et al., 2017). In the graphs are shown the filtered count (left) and the Log‐transformed count (right) for *Shinella fusca* (a) and for the genus *Mesorhizobium* (b).
**Figure S6.** Univariate analysis at phylum level of chickpea bulk soil microbiome. The univariate analysis of the chickpea bulk soil was evaluated comparing the first (July, T0) and the second time point (August, T1) with the web‐based tool Microbiome Analyst (Dhariwal et al., 2017). In the graphs are shown the filtered count (left) and the Log‐transformed count (right) for the phyla Firmicutes (a), Acidobacteria (b), Aquificae (c), Chlorobi (d), and Gemmatimonadetes (e).
**Figure S7.** Univariate analysis at class level of chickpea bulk soil microbiome. The univariate analysis of the chickpea bulk soil was evaluated comparing the first (July, T0) and the second (August, T1) time point with the web‐based tool Microbiome Analyst (Dhariwal et al., 2017). In the graphs are shown the filtered count (left) and the Log‐transformed count (right) for the classes Deltaproteobacteria (a), Alphaproteobacteria (b), Dehalococcidia (c), Clostridia (d), Gloeobacterophycideae (e), Solibacteres (f), Aquificae (g), and Chlorobia (h).
**Figure S8.** Univariate analysis at genus level of chickpea bulk soil microbiome. The univariate analysis of the chickpea bulk soil was evaluated comparing the first (July, T0) and the second (August, T1) time point with the web‐based tool Microbiome Analyst (Dhariwal et al., 2017). In the graphs are shown the filtered count (left) and the Log‐transformed count (right) for the genera *Sphingomonas* (a), *Solirubrobacter* (b), *Lysobacter* (c), *Arenimonas* (d), *Ilumatobacter* (e) and *Glycomyces* (f).
**Figure S9.** Heat tree and pie charts of lentil bulk soil microbiome. The heat tree (a) depicts the hierarchical structure of phyla and the relative abundance of lentil bulk soil microbial communities. The colour gradient and the size of node, edge, and label are based on the log_2_ ratio of median abundance. The pie chart (b) shows the taxonomic abundance of lentil bulk soil. The pie charts (c), (d) show the taxonomic abundance of lentil bulk soil at T0 (c, July) and at T1 (d, August). The graphs were generated with the web‐based tool Microbiome Analyst (Dhariwal et al., 2017).
**Figure S10.** Univariate analysis at class and order level of lentil bulk soil microbiome. The univariate analysis of the lentil bulk soil was evaluated comparing the first (July, T0) and the second time point (August, T1) with the web‐based tool Microbiome Analyst (Dhariwal et al., 2017). In the graphs, the filtered count (left) and the Log‐transformed count (right) are shown for the class Alphaproteobacteria (a), and for the orders Sphingomonadales (b), Gaiellales (c), Rubrobacterales (d), and Clostridiales (e).
**Figure S11.** Univariate analysis at family and genus level of lentil bulk soil microbiome. The univariate analysis of the lentil bulk soil was evaluated comparing the first (July, T0) and the second time point (August, T1) with the web‐based tool Microbiome Analyst (Dhariwal et al., 2017). In the graphs the filtered count (left) and the Log‐transformed count (right) are shown for the families Sphingomonadaceae (a), Gaiellaceae (b), Geobacteraceae (c), Clostridiaceae (d), and Rubrobacteraceae (e), and for the genus Sphingomonas (f).
**Figure S12.** Alpha and beta diversity of lentil bulk soil microbiome. The alpha diversity of the lentil bulk soil microbiome was evaluated at the first (July, T0, a) and at the second time point (August, T1, b) with the Chao1 index, and significant differences were evaluated with ANOVA. The beta diversity among the lentil genotypes (Colfiorito, Elsa, Eston, and Itaca) was evaluated at T0 (c) with the Jaccard index, and significant differences were evaluated with PERMANOVA. The web‐based tool Microbiome Analyst was used (Dhariwal et al., 2017).
**Figure S13.** Lentil parcel showing disease symptoms. In the picture, a suffering lentil parcel of Itaca genotype is reported. Most of the plants in the parcel were dead, the others were suffering, displaying chlorotic leaves.

## Data Availability

The data that support the findings of this study are available from the corresponding author upon reasonable request. Reads have been submitted to NCBI Sequence Read Archive (SRA).
