# Supplementary material for: The ‘microbiome counterattack’: Insights on the soil and root‐associated microbiome in diverse chickpea and lentil genotypes after an erratic rainfall event
Source: Environ Microbiol Rep. 2023 May 24;15(6):459–83. doi: 10.1111/1758-2229.13167 (PMC10667653; doi:10.1111/1758-2229.13167)
Supplement: Supplementary file 1 — Table S1. Climatic data are reported for the period from 01 June 2021 to 30 September 2021. The sowing date was at 04 June 2021. For each day (DD/MM/YYYY), the rain (mm), the average direct solar radiation (Wm−2), the minimum, maximum, and average air temperature (°C), the average relative humidity (%), the wind speed (m s−1) and the reference evapotranspiration (Eto, mm) calculated with FAO Penman–Monteith method and Hargreaves method are reported. [file EMI4-15-459-s003.docx]

**Table S1**. Climatic data are reported for the period from 01/06/2021 to 30/09/2021. The sowing date was at 04/06/2021. For each day (DD/MM/YYYY), the rain (mm), the average direct solar radiation (Wm^-2^), the minimum, maximum and average air temperature (°C), the average relative humidity (%), the wind speed (m s^-1^) and the reference evapotranspiration (Eto, mm) calculated with FAO Penman-Monteith method and Hargreaves method are reported.

| **Date**  **(DD/MM/YYYY)** | **Rain (mm)** | **Direct solar radiation (W m^-2^)_avg** | **Air T (°C)_m in** | **Air T (°C)_max** | **Air T (°C)_avg** | **Relative humidity (%)_avg** | **Wind speed (m s^-1^)_avg** | **Eto (FAO- 56 P&M)**  **(mm)** | **Eto (Hargreaves) (mm)** |
| --- | --- | --- | --- | --- | --- | --- | --- | --- | --- |
| 01/06/2021 | 0 | 303.76 | 10.24 | 26.11 | 18.56 | 48.64 | 3.34 | 6.04 | 5.46 |
| 02/06/2021 | 0 | 329.19 | 8.56 | 25.32 | 17.98 | 46.95 | 1.93 | 5.41 | 5.83 |
| 03/06/2021 | 0 | 332.6 | 8.87 | 28.08 | 19.82 | 45.93 | 1.64 | 5.64 | 6.56 |
| 04/06/2021 | 0 | 325.67 | 12.57 | 29.7 | 22.21 | 52.64 | 1.79 | 5.83 | 6.59 |
| 05/06/2021 | 0 | 231.01 | 14.29 | 29.62 | 21.95 | 62.63 | 1.56 | 4.62 | 6.19 |
| 06/06/2021 | 0.2 | 221.99 | 15.66 | 29.91 | 22.47 | 64.99 | 1.33 | 4.29 | 6.05 |
| 07/06/2021 | 1.19 | 223.78 | 16.64 | 29.8 | 22.14 | 69.5 | 1.5 | 4.55 | 5.76 |
| 08/06/2021 | 0 | 167.64 | 19.26 | 27.1 | 22.37 | 70.25 | 2.52 | 4.04 | 4.48 |
| 09/06/2021 | 0 | 248.33 | 16.18 | 31.43 | 24.03 | 57.62 | 2.07 | 5.43 | 6.5 |
| 10/06/2021 | 0 | 232.55 | 15.79 | 32.71 | 22.39 | 59.66 | 2.76 | 6.03 | 6.58 |
| 11/06/2021 | 7.13 | 199.13 | 17.08 | 31.18 | 22.08 | 62.11 | 3.02 | 5.64 | 5.96 |
| 12/06/2021 | 6.73 | 173.35 | 16.27 | 28.94 | 21.03 | 68.66 | 2.67 | 4.59 | 5.5 |
| 13/06/2021 | 0 | 303.85 | 14.99 | 33.63 | 24.81 | 51.48 | 2.36 | 6.63 | 7.32 |
| 14/06/2021 | 0 | 292.75 | 18.22 | 29.92 | 23.98 | 42.41 | 3.31 | 6.8 | 5.69 |
| 15/06/2021 | 0 | 324.49 | 14.64 | 27.69 | 21.93 | 46.22 | 3.28 | 6.59 | 5.71 |
| 16/06/2021 | 0 | 282.65 | 12.81 | 30.92 | 22.08 | 50.13 | 1.72 | 5.52 | 6.75 |
| 17/06/2021 | 0 | 329.36 | 14.07 | 29.02 | 22.37 | 61.54 | 1.67 | 5.67 | 6.18 |
| 18/06/2021 | 0 | 311 | 14.51 | 30.3 | 23.43 | 62.14 | 1.47 | 5.48 | 6.52 |
| 19/06/2021 | 0 | 308.35 | 17.86 | 34.63 | 27.17 | 51.72 | 1.83 | 6.36 | 7.33 |
| 20/06/2021 | 0 | 294.85 | 19.9 | 36.79 | 28.46 | 52.78 | 1.95 | 6.65 | 7.56 |
| 21/06/2021 | 0 | 272.72 | 20.44 | 34.32 | 27.1 | 67.89 | 1.33 | 5.44 | 6.66 |
| 22/06/2021 | 0 | 264.48 | 21.52 | 35.84 | 27.33 | 70.93 | 1.16 | 5.38 | 6.8 |
| 23/06/2021 | 0 | 292.2 | 20.16 | 34.22 | 27.41 | 72.39 | 1.15 | 5.55 | 6.74 |
| 24/06/2021 | 0 | 205.52 | 21.14 | 38.56 | 29.66 | 62.73 | 1.18 | 4.89 | 7.88 |
| 25/06/2021 | 0 | 187.64 | 21.52 | 39.55 | 30.16 | 44.79 | 1.55 | 5.44 | 8.1 |
| 26/06/2021 | 0 | 300.87 | 21 | 35.54 | 28.55 | 48.05 | 1.8 | 6.52 | 7.03 |
| 27/06/2021 | 0 | 309.56 | 20.23 | 34.63 | 27.73 | 48.8 | 1.44 | 6.17 | 6.87 |
| 28/06/2021 | 0 | 299.39 | 19.32 | 33.48 | 26.68 | 60.36 | 1.5 | 5.87 | 6.66 |
| 29/06/2021 | 0 | 299.8 | 19.04 | 36.31 | 28.1 | 68.59 | 1.84 | 6.41 | 7.59 |
| 30/06/2021 | 0 | 197.35 | 21.25 | 36.66 | 28.83 | 63.99 | 1.64 | 5.06 | 7.28 |
| 01/07/2021 | 0 | 328.72 | 21.59 | 37.77 | 30 | 36.58 | 2.89 | 8.39 | 7.65 |
| 02/07/2021 | 0 | 311.43 | 18.41 | 34.22 | 26.93 | 47.86 | 2.07 | 6.72 | 6.83 |
| 03/07/2021 | 0 | 312.44 | 19.35 | 35.66 | 27.39 | 49.25 | 2.12 | 6.91 | 7.01 |
| 04/07/2021 | 0 | 292.34 | 18.71 | 32.57 | 25.79 | 61.06 | 1.74 | 5.79 | 6.23 |
| 05/07/2021 | 0 | 307.9 | 19.02 | 35.4 | 28.17 | 50.35 | 2.91 | 7.28 | 7.15 |
| 06/07/2021 | 0 | 323.01 | 21.39 | 36.31 | 28.74 | 46.23 | 2.12 | 7.18 | 6.9 |
| 07/07/2021 | 0 | 320.8 | 18.78 | 37.07 | 28.89 | 47.37 | 1.57 | 6.62 | 7.67 |
| 08/07/2021 | 0 | 308.58 | 19.81 | 37.96 | 29.24 | 51.56 | 1.52 | 6.53 | 7.7 |
| 09/07/2021 | 0 | 281.12 | 21.49 | 38.12 | 29.65 | 54.76 | 1.43 | 6.16 | 7.43 |
| 10/07/2021 | 0 | 312.5 | 21.4 | 37.12 | 29.32 | 41.96 | 2 | 7.08 | 7.18 |
| 11/07/2021 | 0 | 303.51 | 19.18 | 34.97 | 27.9 | 49.41 | 1.92 | 6.49 | 6.97 |
| 12/07/2021 | 0 | 307.87 | 21.65 | 35.2 | 28.26 | 54.13 | 1.81 | 6.42 | 6.51 |
| 13/07/2021 | 0 | 277.93 | 20.79 | 33.39 | 27.24 | 73.69 | 1.46 | 5.42 | 6.14 |
| 14/07/2021 | 0 | 294.93 | 23.49 | 37.55 | 29.59 | 56.38 | 2.32 | 7.11 | 6.82 |
| 15/07/2021 | 0 | 311.24 | 19.65 | 33.38 | 27.01 | 43.08 | 2.59 | 7.03 | 6.38 |
| 16/07/2021 | 0 | 278.49 | 17.16 | 32.21 | 25.88 | 50.5 | 2.69 | 6.23 | 6.51 |
| 17/07/2021 | 0.59 | 240.47 | 19.19 | 30.92 | 23.89 | 66.19 | 2.06 | 5.13 | 5.48 |
| 18/07/2021 | 26.93 | 184.03 | 18.28 | 30.65 | 21.81 | 78.99 | 2.8 | 4.76 | 5.35 |
| 19/07/2021 | 3.17 | 146.47 | 18.82 | 30.54 | 22.76 | 81.91 | 2.24 | 3.91 | 5.33 |
| 20/07/2021 | 0 | 187.79 | 19.89 | 31.57 | 25.36 | 70.51 | 1.94 | 4.34 | 5.67 |
| 21/07/2021 | 0 | 312.55 | 19.64 | 34.28 | 27.24 | 47.16 | 2.85 | 7.13 | 6.62 |
| 22/07/2021 | 0 | 305.09 | 17.97 | 34.65 | 26.77 | 45.43 | 2.93 | 7.22 | 6.99 |
| 23/07/2021 | 0 | 299.4 | 17.87 | 33.51 | 26.03 | 50.74 | 1.51 | 5.82 | 6.66 |
| 24/07/2021 | 0 | 286.71 | 18.51 | 34.81 | 26.65 | 60.15 | 1.52 | 5.72 | 6.89 |
| 25/07/2021 | 0 | 284.76 | 18.58 | 32.82 | 26.23 | 71.04 | 1.47 | 5.41 | 6.38 |
| 26/07/2021 | 0 | 281.44 | 20.64 | 34.98 | 27.25 | 74.57 | 1.35 | 5.54 | 6.55 |
| 27/07/2021 | 0 | 284.21 | 21.04 | 35.79 | 28.05 | 73.27 | 1.31 | 5.65 | 6.76 |
| 28/07/2021 | 0 | 283.74 | 22.32 | 38.41 | 30.17 | 50.39 | 1.31 | 6.05 | 7.39 |
| 29/07/2021 | 0 | 293.06 | 20.44 | 41.07 | 30.36 | 47.33 | 1.59 | 6.79 | 8.4 |
| 30/07/2021 | 0 | 291.1 | 21.67 | 38.55 | 29.72 | 46.55 | 1.83 | 6.74 | 7.5 |
| 31/07/2021 | 0 | 298.16 | 20.6 | 37.61 | 28.99 | 52.36 | 1.65 | 6.41 | 7.41 |
| 01/08/2021 | 0 | 281.27 | 20.51 | 37.75 | 29.33 | 57.46 | 1.53 | 6.03 | 7.52 |
| 02/08/2021 | 0 | 270.32 | 23.73 | 37.65 | 30.88 | 48.81 | 2.3 | 6.63 | 6.35 |
| 03/08/2021 | 0 | 255.54 | 21.3 | 36.55 | 28.46 | 45.25 | 2.29 | 6.47 | 6.31 |
| 04/08/2021 | 0 | 261.88 | 20.97 | 32.7 | 27.34 | 62.42 | 1.72 | 5.27 | 5.4 |
| 05/08/2021 | 0 | 154.88 | 23.61 | 35.76 | 28.66 | 56.35 | 2.96 | 5.43 | 5.66 |
| 06/08/2021 | 0 | 280.07 | 19.02 | 34.01 | 27.23 | 45.94 | 2.23 | 6.36 | 6.1 |
| 07/08/2021 | 0 | 293.22 | 19.72 | 33.6 | 26.85 | 56.88 | 1.73 | 5.81 | 5.82 |
| 08/08/2021 | 0 | 281.24 | 19.4 | 33.46 | 27.03 | 72.15 | 1.39 | 5.25 | 5.88 |
| 09/08/2021 | 0 | 272.25 | 21.88 | 36.79 | 29.12 | 62.33 | 1.74 | 5.96 | 6.33 |
| 10/08/2021 | 0 | 265.98 | 21.06 | 39.88 | 30.54 | 49.03 | 1.87 | 6.41 | 7.33 |
| 11/08/2021 | 0 | 198.65 | 24.65 | 40.36 | 31.08 | 46.73 | 2.04 | 5.99 | 6.77 |
| 12/08/2021 | 0 | 276.85 | 23.49 | 37.6 | 30.57 | 42.77 | 2.59 | 7.08 | 6.35 |
| 13/08/2021 | 0 | 272.66 | 21.55 | 39.38 | 30.06 | 45.28 | 1.73 | 6.42 | 7.07 |
| 14/08/2021 | 0 | 274.8 | 20.56 | 39.12 | 29.84 | 40.4 | 1.74 | 6.36 | 7.18 |
| 15/08/2021 | 0 | 276.21 | 18.95 | 37.47 | 27.83 | 46.98 | 1.65 | 6.03 | 6.87 |
| 16/08/2021 | 0 | 268.25 | 18.88 | 35.81 | 27.1 | 67.9 | 1.29 | 5.24 | 6.46 |
| 17/08/2021 | 0 | 252.62 | 22.21 | 36.5 | 28.16 | 76.3 | 1.5 | 5.42 | 6.07 |
| 18/08/2021 | 0 | 195.38 | 23.55 | 34.16 | 28.09 | 59.41 | 2.55 | 5.25 | 5.23 |
| 19/08/2021 | 0 | 271.43 | 21.94 | 34.68 | 27.78 | 48.77 | 2.42 | 6.35 | 5.69 |
| 20/08/2021 | 0 | 261.38 | 18.37 | 33.03 | 26.33 | 52.54 | 2.27 | 5.7 | 5.91 |
| 21/08/2021 | 0 | 265.96 | 19.52 | 34.74 | 26.71 | 52.64 | 2.29 | 6.07 | 6.07 |
| 22/08/2021 | 0 | 264.53 | 17.19 | 35.69 | 26.94 | 47.74 | 1.62 | 5.54 | 6.73 |
| 23/08/2021 | 0 | 258.23 | 19.17 | 34.27 | 26.69 | 60.09 | 1.65 | 5.22 | 6.04 |
| 24/08/2021 | 0 | 252.67 | 21.21 | 34.34 | 27.39 | 64.69 | 1.92 | 5.41 | 5.72 |
| 25/08/2021 | 18.81 | 188.73 | 21.67 | 31.7 | 25.32 | 75.05 | 1.84 | 4.03 | 4.77 |
| 26/08/2021 | 17.42 | 159.15 | 18.28 | 29.7 | 22.75 | 87.64 | 1.46 | 3.14 | 4.79 |
| 27/08/2021 | 0.2 | 256.81 | 17.63 | 31.64 | 24.91 | 71.2 | 1.32 | 4.53 | 5.59 |
| 28/08/2021 | 0 | 216.25 | 18.43 | 30.64 | 24.41 | 67.9 | 2.16 | 4.48 | 5.16 |
| 29/08/2021 | 0 | 154.51 | 17.47 | 27.78 | 22.27 | 72.45 | 2.2 | 3.53 | 4.5 |
| 30/08/2021 | 0 | 242.92 | 17.04 | 27.92 | 22.38 | 65.28 | 1.95 | 4.36 | 4.63 |
| 31/08/2021 | 0 | 262.05 | 15.68 | 30.54 | 23.2 | 63.49 | 1.39 | 4.56 | 5.53 |
| 01/09/2021 | 0 | 211.32 | 17.71 | 29.6 | 23.33 | 63.68 | 2.22 | 4.34 | 4.96 |
| 02/09/2021 | 0 | 251.61 | 18.29 | 29.32 | 23.39 | 55.93 | 3.38 | 5.65 | 3.93 |
| 03/09/2021 | 0 | 210.55 | 16.67 | 29.64 | 22.67 | 56.73 | 2.06 | 4.45 | 4.19 |
| 04/09/2021 | 0 | 175.1 | 20.03 | 29.91 | 23.87 | 70.95 | 1.63 | 3.72 | 3.77 |
| 05/09/2021 | 8.51 | 155.49 | 19.58 | 27.98 | 22.27 | 83.81 | 1.58 | 3.03 | 3.34 |
| 06/09/2021 | 17.03 | 141.11 | 17.66 | 29.27 | 21.32 | 81.66 | 2.38 | 3.48 | 3.83 |
| 07/09/2021 | 1.19 | 176.18 | 16.44 | 28.85 | 20.97 | 74.87 | 2.27 | 3.96 | 3.93 |
| 08/09/2021 | 2.77 | 140.51 | 14.85 | 27.9 | 19.43 | 79.67 | 1.98 | 3.4 | 3.87 |
| 09/09/2021 | 0 | 190.24 | 14.39 | 27.9 | 20.23 | 73.75 | 1.08 | 3.25 | 4.02 |
| 10/09/2021 | 0 | 228.02 | 14.15 | 27.35 | 20.67 | 75.06 | 1.31 | 3.59 | 4.02 |
| 11/09/2021 | 12.87 | 75.84 | 17.57 | 23.76 | 19.86 | 88.45 | 1.37 | 1.72 | 2.69 |
| 12/09/2021 | 2.77 | 124.18 | 17.21 | 27.78 | 20.75 | 81.88 | 1.48 | 2.73 | 3.6 |
| 13/09/2021 | 0 | 231.89 | 15.42 | 30.83 | 22.78 | 65.05 | 1.48 | 4.16 | 4.58 |
| 14/09/2021 | 0 | 226.7 | 14.99 | 31.3 | 22.71 | 64.48 | 1.34 | 4.05 | 4.7 |
| 15/09/2021 | 0 | 204.53 | 15.42 | 31.4 | 23 | 66.85 | 1.2 | 3.71 | 4.69 |
| 16/09/2021 | 0 | 201.24 | 18.42 | 29.85 | 23.86 | 71.79 | 1.27 | 3.56 | 4.05 |
| 17/09/2021 | 0.2 | 145.1 | 20.24 | 29.59 | 24.23 | 85.78 | 1.46 | 2.95 | 3.69 |
| 18/09/2021 | 0.4 | 150.37 | 20 | 31.64 | 24.36 | 77.13 | 1.31 | 3.15 | 4.14 |
| 19/09/2021 | 0 | 206.24 | 19.18 | 30.7 | 24.63 | 73.17 | 1.61 | 3.79 | 4.14 |
| 20/09/2021 | 0 | 190.5 | 19.5 | 30.91 | 24.71 | 62.84 | 1.57 | 3.84 | 4.13 |
| 21/09/2021 | 0 | 184.5 | 16.27 | 30.46 | 23.24 | 61.8 | 2.18 | 4.18 | 4.44 |
| 22/09/2021 | 0 | 196.86 | 17.09 | 27.04 | 21.39 | 54.12 | 4.31 | 5.18 | 3.55 |
| 23/09/2021 | 0 | 203.53 | 13.29 | 24.97 | 19.47 | 52.88 | 3.36 | 4.33 | 3.66 |
| 24/09/2021 | 0 | 221.97 | 9.32 | 25.44 | 17.51 | 64.07 | 1.42 | 3.29 | 4.08 |
| 25/09/2021 | 0 | 210.71 | 11.87 | 27.81 | 19.53 | 74.39 | 1.25 | 3.17 | 4.28 |
| 26/09/2021 | 0 | 190.24 | 13.63 | 27.36 | 20.65 | 79.65 | 1.17 | 2.91 | 4.1 |
| 27/09/2021 | 0.2 | 102.8 | 15.6 | 28.64 | 21.67 | 83.49 | 1.27 | 2.37 | 4.1 |
| 28/09/2021 | 0 | 144.41 | 19.8 | 29.42 | 23.98 | 69.99 | 2.87 | 3.51 | 3.73 |
| 29/09/2021 | 0 | 179.36 | 15.72 | 29.6 | 22.33 | 64.2 | 1.75 | 3.56 | 4.3 |
| 30/09/2021 | 0 | 162.15 | 15.24 | 28.84 | 21.39 | 72.61 | 1.33 | 2.89 | 4.15 |
