# Supplementary material for: The ‘microbiome counterattack’: Insights on the soil and root‐associated microbiome in diverse chickpea and lentil genotypes after an erratic rainfall event
Source: Environ Microbiol Rep. 2023 May 24;15(6):459–83. doi: 10.1111/1758-2229.13167 (PMC10667653; doi:10.1111/1758-2229.13167)
Supplement: Supplementary file 3 — Table S6. List of prokaryotic families specific and shared among lentil roots, bulk soil at T0 (July), and bulk soil at T1 (August). The ‘Compartment(s)’ column indicates the specific or shared sample type, the ‘Number of elements’ column indicates the number of prokaryotic families found in each compartment, and in the ‘Families’ column the prokaryotic families are listed. The Venn diagram table was obtained through the https://bioinformatics.psb.ugent.be/ web‐based tool. [file EMI4-15-459-s002.docx]

**Table S6.** List of prokaryotic families specific and shared among lentil roots, bulk soil at T0 (July) and bulk soil at T1 (August). The “Compartment(s)” column indicates the specific or shared sample type, the “Number of elements” column indicates the number of prokaryotic families found in each compartment, and in the “Families” column the prokaryotic families are listed. The Venn diagram table was obtained through the <https://bioinformatics.psb.ugent.be/> web-based tool.

| **Compartment(s)** | | **Number of elements** | **Families** |
| --- | --- | --- | --- |
| Bulk T0 Bulk T1 Roots | 54 | | f_Anaerolineaceae |
|  |  |  | f_Bacillaceae |
|  |  |  | f_Bdellovibrionaceae |
|  |  |  | f_Beijerinckiaceae |
|  |  |  | f_Burkholderiaceae |
|  |  |  | f_Caldilineaceae |
|  |  |  | f_Caulobacteraceae |
|  |  |  | f_Chitinophagaceae |
|  |  |  | f_Clostridiaceae |
|  |  |  | f_Comamonadaceae |
|  |  |  | f_Cyclobacteriaceae |
|  |  |  | f_Cytophagaceae |
|  |  |  | f_Enterobacteriaceae |
|  |  |  | f_Flavobacteriaceae |
|  |  |  | f_Gaiellaceae |
|  |  |  | f_Gemmatimonadaceae |
|  |  |  | f_Geodermatophilaceae |
|  |  |  | f_Herpetosiphonaceae |
|  |  |  | f_Hyphomicrobiaceae |
|  |  |  | f_Hyphomonadaceae |
|  |  |  | f_Iamiaceae |
|  |  |  | f_Intrasporangiaceae |
|  |  |  | f_Microbacteriaceae |
|  |  |  | f_Micrococcaceae |
|  |  |  | f_Micromonosporaceae |
|  |  |  | f_Moraxellaceae |
|  |  |  | f_Mycobacteriaceae |
|  |  |  | f_Myxococcaceae |
|  |  |  | f_Nitrosomonadaceae |
|  |  |  | f_Nitrospiraceae |
|  |  |  | f_Nocardiaceae |
|  |  |  | f_Nocardioidaceae |
|  |  |  | f_Nostocaceae |
|  |  |  | f_Opitutaceae |
|  |  |  | f_Oxalobacteraceae |
|  |  |  | f_Paenibacillaceae |
|  |  |  | f_Peptostreptococcaceae |
|  |  |  | f_Promicromonosporaceae |
|  |  |  | f_Pseudomonadaceae |
|  |  |  | f_Pseudonocardiaceae |
|  |  |  | f_Rhizobiaceae |
|  |  |  | f_Rhodobacteraceae |
|  |  |  | f_Rhodocyclaceae |
|  |  |  | f_Roseiflexaceae |
|  |  |  | f_Sandaracinaceae |
|  |  |  | f_Solirubrobacteraceae |
|  |  |  | f_Sphingobacteriaceae |
|  |  |  | f_Sphingomonadaceae |
|  |  |  | f_Streptomycetaceae |
|  |  |  | f_Streptosporangiaceae |
|  |  |  | f_Thermoactinomycetaceae |
|  |  |  | f_Thermomonosporaceae |
|  |  |  | f_Verrucomicrobiaceae |
|  |  |  | f_Xanthomonadaceae |
| Bulk T0  Roots | 2 | | f_Glycomycetaceae |
|  |  |  | f_Phaselicystidaceae |
| Bulk T1  Roots | 5 | | f_Alcaligenaceae |
|  |  |  | f_Alicyclobacillaceae |
|  |  |  | f_Planococcaceae |
|  |  |  | f_Polyangiaceae |
|  |  |  | f_Saprospiraceae |
| Bulk T0  Bulk T1 | 78 | | f_Acanthopleuribacteraceae |
|  |  |  | f_Acetobacteraceae |
|  |  |  | f_Acidimicrobiaceae |
|  |  |  | f_Acidithiobacillaceae |
|  |  |  | f_Acidobacteriaceae |
|  |  |  | f_Acidothermaceae |
|  |  |  | f_Alcanivoracaceae |
|  |  |  | f_Aquificaceae |
|  |  |  | f_Bacteroidaceae |
|  |  |  | f_Bartonellaceae |
|  |  |  | f_Bradyrhizobiaceae |
|  |  |  | f_Cellulomonadaceae |
|  |  |  | f_Chlorobiaceae |
|  |  |  | f_Chromatiaceae |
|  |  |  | f_Conexibacteraceae |
|  |  |  | f_Coriobacteriaceae |
|  |  |  | f_Cystobacteraceae |
|  |  |  | f_Dehalococcoidaceae |
|  |  |  | f_Desulfobulbaceae |
|  |  |  | f_Desulfohalobiaceae |
|  |  |  | f_Desulfonatronumaceae |
|  |  |  | f_Desulfovibrionaceae |
|  |  |  | f_Desulfurellaceae |
|  |  |  | f_Desulfuromonadaceae |
|  |  |  | f_Ectothiorhodospiraceae |
|  |  |  | f_Enterococcaceae |
|  |  |  | f_Erythrobacteraceae |
|  |  |  | f_Euzebyaceae |
|  |  |  | f_Flammeovirgaceae |
|  |  |  | f_Frankiaceae |
|  |  |  | f_Geminicoccus |
|  |  |  | f_Geobacteraceae |
|  |  |  | f_Gloeobacteraceae |
|  |  |  | f_Halomonadaceae |
|  |  |  | f_Holophagaceae |
|  |  |  | f_Hydrogenophilaceae |
|  |  |  | f_Jiangellaceae |
|  |  |  | f_Kineosporiaceae |
|  |  |  | f_Kofleriaceae |
|  |  |  | f_Koribacteraceae |
|  |  |  | f_Methylobacteriaceae |
|  |  |  | f_Methylococcaceae |
|  |  |  | f_Methylocystaceae |
|  |  |  | f_Nitrososphaeraceae |
|  |  |  | f_Nitrospinaceae |
|  |  |  | f_Oceanospirillaceae |
|  |  |  | f_Oscillochloridaceae |
|  |  |  | f_Pelobacteraceae |
|  |  |  | f_Peptococcaceae |
|  |  |  | f_Phyllobacteriaceae |
|  |  |  | f_Planctomycetaceae |
|  |  |  | f_Propionibacteriaceae |
|  |  |  | f_Rhodospirillaceae |
|  |  |  | f_Rhodothermaceae |
|  |  |  | f_Rubrobacteraceae |
|  |  |  | f_Ruminococcaceae |
|  |  |  | f_Sanguibacteraceae |
|  |  |  | f_Sinobacteraceae |
|  |  |  | f_Solibacteraceae |
|  |  |  | f_Sphaerobacteraceae |
|  |  |  | f_Spirochaetaceae |
|  |  |  | f_Sporichthyaceae |
|  |  |  | f_Streptococcaceae |
|  |  |  | f_Synergistaceae |
|  |  |  | f_Syntrophaceae |
|  |  |  | f_Syntrophobacteraceae |
|  |  |  | f_Syntrophomonadaceae |
|  |  |  | f_Thermoanaerobacteraceae |
|  |  |  | f_Thermoleophilaceae |
|  |  |  | f_Thermolithobacteraceae |
|  |  |  | f_Thiohalorhabdus |
|  |  |  | f_Thiotrichaceae |
|  |  |  | f_Tsukamurellaceae |
|  |  |  | f_unclassifiedBurkholderiales |
|  |  |  | f_unclassifiedDehalococcoidia |
|  |  |  | f_unclassifiedRhizobiales |
|  |  |  | f_Veillonellaceae |
|  |  |  | f_Verrucomicrobiasubdivision3 |
| Roots | 90 | | f_01D2Z36 |
|  |  |  | f_0319-6G20 |
|  |  |  | f_0319-7L14 |
|  |  |  | f_67-14 |
|  |  |  | f_A4b |
|  |  |  | f_Abditibacteriaceae |
|  |  |  | f_AKIW781 |
|  |  |  | f_AKYG1722 |
|  |  |  | f_Ardenticatenaceae |
|  |  |  | f_Azospirillaceae |
|  |  |  | f_bacteriap25 |
|  |  |  | f_Bacteriovoracaceae |
|  |  |  | f_Bacteroidetes_VC2.1_Bac22 |
|  |  |  | f_BIrii41 |
|  |  |  | f_Blastocatellaceae |
|  |  |  | f_Bryobacteraceae |
|  |  |  | f_C0119 |
|  |  |  | f_Candidatus_Pacebacteria |
|  |  |  | f_CCD24 |
|  |  |  | f_Cellvibrionaceae |
|  |  |  | f_Chloroflexaceae |
|  |  |  | f_Chthoniobacteraceae |
|  |  |  | f_Crocinitomicaceae |
|  |  |  | f_Dermabacteraceae |
|  |  |  | f_Devosiaceae |
|  |  |  | f_Diplorickettsiaceae |
|  |  |  | f_Dongiaceae |
|  |  |  | f_Entotheonellaceae |
|  |  |  | f_Erwiniaceae |
|  |  |  | f_Exiguobacteraceae |
|  |  |  | f_Ferrovibrionaceae |
|  |  |  | f_Fimbriimonadaceae |
|  |  |  | f_Geminicoccaceae |
|  |  |  | f_Gemmataceae |
|  |  |  | f_Gitt-GS-136 |
|  |  |  | f_Haliangiaceae |
|  |  |  | f_Hymenobacteraceae |
|  |  |  | f_Ilumatobacteraceae |
|  |  |  | f_IMCC26256 |
|  |  |  | f_JG30-KF-CM45 |
|  |  |  | f_KD4-96 |
|  |  |  | f_Legionellaceae |
|  |  |  | f_LWQ8 |
|  |  |  | f_MB-A2-108 |
|  |  |  | f_Methylophilaceae |
|  |  |  | f_Microscillaceae |
|  |  |  | f_Nannocystaceae |
|  |  |  | f_Nitrosococcaceae |
|  |  |  | f_Nodosilineaceae |
|  |  |  | f_NS11-12_marine_group |
|  |  |  | f_Obscuribacteraceae |
|  |  |  | f_Oscillatoriaceae |
|  |  |  | f_Parachlamydiaceae |
|  |  |  | f_Pedosphaeraceae |
|  |  |  | f_Phormidiaceae |
|  |  |  | f_Pirellulaceae |
|  |  |  | f_Puniceicoccaceae |
|  |  |  | f_Pyrinomonadaceae |
|  |  |  | f_Reyranellaceae |
|  |  |  | f_Rhodanobacteraceae |
|  |  |  | f_Rubinisphaeraceae |
|  |  |  | f_Rubritaleaceae |
|  |  |  | f_Rubrobacteriaceae |
|  |  |  | f_S085 |
|  |  |  | f_Saccharimonadaceae |
|  |  |  | f_Saccharimonadales |
|  |  |  | f_Sericytochromatia |
|  |  |  | f_SM2D12 |
|  |  |  | f_Spirosomaceae |
|  |  |  | f_Steroidobacteraceae |
|  |  |  | f_Sumerlaeaceae |
|  |  |  | f_Sutterellaceae |
|  |  |  | f_Tepidisphaeraceae |
|  |  |  | f_Tepidisphaerales |
|  |  |  | f_Terrimicrobiaceae |
|  |  |  | f_Thermoanaerobaculaceae |
|  |  |  | f_TK10 |
|  |  |  | f_TRA3-20 |
|  |  |  | f_Trueperaceae |
|  |  |  | f_uncultured |
|  |  |  | f_Unknown_Family |
|  |  |  | f_Vampirovibrionaceae |
|  |  |  | f_Vampirovibrionales |
|  |  |  | f_Vermiphilaceae |
|  |  |  | f_Vicinamibacteraceae |
|  |  |  | f_WD2101_soil_group |
|  |  |  | f_Weeksellaceae |
|  |  |  | f_Xanthobacteraceae |
|  |  |  | o_Nitrososphaerales |
| Bulk T0 | 5 | | f_Desulfobacteraceae |
|  |  |  | f_Lachnospiraceae |
|  |  |  | f_Neisseriaceae |
|  |  |  | f_Rhabdochlamydiaceae |
|  |  |  | f_Thermaceae |
| Bulk T1 | 12 | | f_Aciditerrimonas |
|  |  |  | f_Alteromonadaceae |
|  |  |  | f_Anaplasmataceae |
|  |  |  | f_Ardenscatenaceae |
|  |  |  | f_Leuconostocaceae |
|  |  |  | f_Microthrixaceae |
|  |  |  | f_Nitriliruptoraceae |
|  |  |  | f_Patulibacteraceae |
|  |  |  | f_Proteinivoraceae |
|  |  |  | f_Segniliparaceae |
|  |  |  | f_unclassifiedGammaproteobacteria |
|  |  |  | f_unclassifiedRhodospirillales |
