# Supplementary material for: The ‘microbiome counterattack’: Insights on the soil and root‐associated microbiome in diverse chickpea and lentil genotypes after an erratic rainfall event
Source: Environ Microbiol Rep. 2023 May 24;15(6):459–83. doi: 10.1111/1758-2229.13167 (PMC10667653; doi:10.1111/1758-2229.13167)
Supplement: Supplementary file 4 — Table S7. List of prokaryotic families specific and shared among lentil root samples, bulk soil at T1 (August). The ‘Compartment(s)’ column indicates the specific or shared sample type, the ‘Number of elements’ column indicates the number of prokaryotic families found in each compartment, and in the ‘Families’ column the prokaryotic families are listed. The Venn diagram table was obtained through the https://bioinformatics.psb.ugent.be/ web‐based tool. [file EMI4-15-459-s005.docx]

**Table S7**. List of prokaryotic families specific and shared among lentil root samples, bulk soil at T1 (August). The “Compartment(s)” column indicates the specific or shared sample type, the “Number of elements” column indicates the number of prokaryotic families found in each compartment, and in the “Families” column the prokaryotic families are listed. The Venn diagram table was obtained through the https://bioinformatics.psb.ugent.be/ web-based tool.

| **Compartment(s)** | **Number of elements** | **Families** |
| --- | --- | --- |
| Bulk_T1 Colfiorito Elsa Eston Itaca | 40 | f_ Moraxellaceae |
|  |  | f_ Mycobacteriaceae |
|  |  | f_ Beijerinckiaceae |
|  |  | f_ Nocardiaceae |
|  |  | f_ Oxalobacteraceae |
|  |  | f_ Verrucomicrobiaceae |
|  |  | f_ Myxococcaceae |
|  |  | f_ Roseiflexaceae |
|  |  | f_ Rhodobacteraceae |
|  |  | f_ Solirubrobacteraceae |
|  |  | f_ Cyclobacteriaceae |
|  |  | f_ Streptomycetaceae |
|  |  | f_ Sphingomonadaceae |
|  |  | f_ Intrasporangiaceae |
|  |  | f_ Chitinophagaceae |
|  |  | f_ Micromonosporaceae |
|  |  | f_ Promicromonosporaceae |
|  |  | f_ Caulobacteraceae |
|  |  | f_ Comamonadaceae |
|  |  | f_ Paenibacillaceae |
|  |  | f_ Nocardioidaceae |
|  |  | f_ Bdellovibrionaceae |
|  |  | f_ Rhizobiaceae |
|  |  | f_ Pseudonocardiaceae |
|  |  | f_ Alcaligenaceae |
|  |  | f_ Micrococcaceae |
|  |  | f_ Xanthomonadaceae |
|  |  | f_ Pseudomonadaceae |
|  |  | f_ Cytophagaceae |
|  |  | f_ Planococcaceae |
|  |  | f_ Gemmatimonadaceae |
|  |  | f_ Flavobacteriaceae |
|  |  | f_ Opitutaceae |
|  |  | f_ Sphingobacteriaceae |
|  |  | f_ Microbacteriaceae |
|  |  | f_ Rhodocyclaceae |
|  |  | f_ Enterobacteriaceae |
|  |  | f_ Bacillaceae |
|  |  | f_ Nitrospiraceae |
|  |  | f_ Rubrobacteraceae |
| Colfiorito Elsa Eston Itaca | 39 |  |
|  |  | f_ uncultured |
|  |  | f_ Steroidobacteraceae |
|  |  | f_ AKIW781 |
|  |  | f_ Azospirillaceae |
|  |  | f_ Xanthobacteraceae |
|  |  | f_ Pirellulaceae |
|  |  | f_ Saccharimonadales |
|  |  | f_ Cellvibrionaceae |
|  |  | f_ Weeksellaceae |
|  |  | f_ Chloroflexaceae |
|  |  | f_ TK10 |
|  |  | f_ MB-A2-108 |
|  |  | f_ bacteriap25 |
|  |  | f_ AKYG1722 |
|  |  | f_ Chthoniobacteraceae |
|  |  | f_ Rubinisphaeraceae |
|  |  | f_ Devosiaceae |
|  |  | f_ KD4-96 |
|  |  | f_ Vicinamibacteraceae |
|  |  | f_ Microscillaceae |
|  |  | f_ Methylophilaceae |
|  |  | f_ Ferrovibrionaceae |
|  |  | f_ Thermoanaerobaculaceae |
|  |  | f_ 67-14 |
|  |  | f_ LWQ8 |
|  |  | f_ Blastocatellaceae |
|  |  | f_ A4b |
|  |  | f_ Saccharimonadaceae |
|  |  | f_ Ilumatobacteraceae |
|  |  | f_ Glycomycetaceae |
|  |  | f_ Dongiaceae |
|  |  | f_ Bryobacteraceae |
|  |  | f_ JG30-KF-CM45 |
|  |  | f_ Spirosomaceae |
|  |  | f_ S085 |
|  |  | f_ Hymenobacteraceae |
|  |  | f_ Crocinitomicaceae |
|  |  | f_ Rubritaleaceae |
| Bulk_T1 Colfiorito Elsa Itaca | 4 | f_ Anaerolineaceae |
|  |  | f_ Thermoactinomycetaceae |
|  |  | f_ Sandaracinaceae |
|  |  | f_ Caldilineaceae |
| Bulk_T1 Elsa Eston Itaca | 1 | f_ Streptosporangiaceae |
| Colfiorito Elsa Eston | 7 | f_ 0319-7L14 |
|  |  | f_ OLB14 |
|  |  | f_ Parachlamydiaceae |
|  |  | f_ WD2101_soil_group |
|  |  | f_ Unknown_Family |
|  |  | f_ TRA3-20 |
|  |  | f_ Rhodanobacteraceae |
| Colfiorito Elsa Itaca | 6 | f_ Sericytochromatia |
|  |  | f_ Pyrinomonadaceae |
|  |  | f_ Sumerlaeaceae |
|  |  | f_ Reyranellaceae |
|  |  | f_ Gemmataceae |
|  |  | f_ Geminicoccaceae |
| Bulk_T1 Colfiorito Elsa | 2 | f_ Nostocaceae |
|  |  | f_ Nitrosomonadaceae |
| Colfiorito Eston Itaca | 3 | f_ Pedosphaeraceae |
|  |  | f_ Tepidisphaeraceae |
|  |  | f_ Abditibacteriaceae |
| Bulk_T1 Colfiorito Itaca | 2 | f_ Herpetosiphonaceae |
|  |  | f_ Saprospiraceae |
| Elsa Eston Itaca | 2 | f_ Dermabacteraceae |
|  |  | f_ Erwiniaceae |
| Bulk_T1 Elsa Itaca | 4 | f_ Geodermatophilaceae |
|  |  | f_ Peptostreptococcaceae |
|  |  | f_ Thermomonosporaceae |
|  |  | f_ Clostridiaceae |
| Colfiorito Elsa | 3 | f_ Nannocystaceae |
|  |  | f_ S0134_terrestrial_group |
|  |  | f_ DS-100 |
| Colfiorito Eston | 4 | f_ Exiguobacteraceae |
|  |  | f_ Fimbriimonadaceae |
|  |  | f_ NS11-12_marine_group |
|  |  | f_ Phormidiaceae |
| Colfiorito Itaca | 2 | f_ Ardenticatenaceae |
|  |  | f_ BIrii41 |
| Bulk_T1 Colfiorito | 3 | f_ Burkholderiaceae |
|  |  | f_ Nitrososphaeraceae |
|  |  | f_ Hyphomonadaceae |
| Elsa Eston | 1 | f_ Vampirovibrionales |
| Elsa Itaca | 6 | f_ Gitt-GS-136 |
|  |  | f_ C0119 |
|  |  | f_ Legionellaceae |
|  |  | f_ IMCC26256 |
|  |  | f_ Entotheonellaceae |
|  |  | f_ CCD24 |
| Bulk_T1 Elsa | 4 | f_ Hyphomicrobiaceae |
|  |  | f_ Iamiaceae |
|  |  | f_ Gaiellaceae |
|  |  | f_ Polyangiaceae |
| Eston Itaca | 3 | f_ Haliangiaceae |
|  |  | f_ Trueperaceae |
|  |  | f_ SM2D12 |
| Bulk_T1 Itaca | 1 | f_ Alicyclobacillaceae |
| Colfiorito | 11 | f_ Nodosilineaceae |
|  |  | f_ Puniceicoccaceae |
|  |  | f_ Candidatus_Pacebacteria |
|  |  | f_ Oscillatoriaceae |
|  |  | f_ Bacteroidetes_VC2.1_Bac22 |
|  |  | f_ Phaselicystidaceae |
|  |  | f_ Diplorickettsiaceae |
|  |  | f_ Vermiphilaceae |
|  |  | f_ Bacteriovoracaceae |
|  |  | f_ Sutterellaceae |
|  |  | f_ Terrimicrobiaceae |
| Elsa | 3 | f_ 0319-6G20 |
|  |  | f_ Nitrosococcaceae |
|  |  | f_ Tepidisphaerales |
| Eston | 2 | f_ Obscuribacteraceae |
|  |  | f_ SBR1031 |
| Itaca | 6 | f_ WD2101_ soil_ group |
|  |  | f_ 01D2Z36 |
|  |  | f_ Vampirovibrionaceae |
|  |  | f_ DEV007 |
|  |  | f_ S0134_ terrestrial_ group |
|  |  | f_ Unknown_ Family |
| Bulk_T1 | 88 | f_ Acidithiobacillaceae |
|  |  | f_ Desulfonatronumaceae |
|  |  | f_ Methylobacteriaceae |
|  |  | f_ Ruminococcaceae |
|  |  | f_ Euzebyaceae |
|  |  | f_ Tsukamurellaceae |
|  |  | f_ Bacteroidaceae |
|  |  | f_ Thiohalorhabdus |
|  |  | f_ Frankiaceae |
|  |  | f_ Oscillochloridaceae |
|  |  | f_ unclassifiedGammaproteobacteria |
|  |  | f_ Acidimicrobiaceae |
|  |  | f_ Rhodospirillaceae |
|  |  | f_ Desulfobulbaceae |
|  |  | f_ Desulfohalobiaceae |
|  |  | f_ Aciditerrimonas |
|  |  | f_ Geobacteraceae |
|  |  | f_ Patulibacteraceae |
|  |  | f_ Nitriliruptoraceae |
|  |  | f_ Ectothiorhodospiraceae |
|  |  | f_ Jiangellaceae |
|  |  | f_ Chromatiaceae |
|  |  | f_ Flammeovirgaceae |
|  |  | f_ Streptococcaceae |
|  |  | f_ Geminicoccus |
|  |  | f_ Sporichthyaceae |
|  |  | f_ Sinobacteraceae |
|  |  | f_ Syntrophaceae |
|  |  | f_ Methylocystaceae |
|  |  | f_ Aquificaceae |
|  |  | f_ Alcanivoracaceae |
|  |  | f_ Hydrogenophilaceae |
|  |  | f_ Kineosporiaceae |
|  |  | f_ Conexibacteraceae |
|  |  | f_ Desulfovibrionaceae |
|  |  | f_ Methylococcaceae |
|  |  | f_ Cellulomonadaceae |
|  |  | f_ Phyllobacteriaceae |
|  |  | f_ unclassifiedBurkholderiales |
|  |  | f_ Anaplasmataceae |
|  |  | f_ Planctomycetaceae |
|  |  | f_ Erythrobacteraceae |
|  |  | f_ Kofleriaceae |
|  |  | f_ Sphaerobacteraceae |
|  |  | f_ Acidobacteriaceae |
|  |  | f_ Oceanospirillaceae |
|  |  | f_ Rhodothermaceae |
|  |  | f_ unclassifiedDehalococcoidia |
|  |  | f_ Ardenscatenaceae |
|  |  | f_ unclassifiedRhodospirillales |
|  |  | f_ Acanthopleuribacteraceae |
|  |  | f_ Peptococcaceae |
|  |  | f_ Spirochaetaceae |
|  |  | f_ Verrucomicrobiasubdivision3 |
|  |  | f_ Gloeobacteraceae |
|  |  | f_ Veillonellaceae |
|  |  | f_ Holophagaceae |
|  |  | f_ unclassifiedRhizobiales |
|  |  | f_ Acetobacteraceae |
|  |  | f_ Syntrophomonadaceae |
|  |  | f_ Pelobacteraceae |
|  |  | f_ Desulfurellaceae |
|  |  | f_ Acidothermaceae |
|  |  | f_ Halomonadaceae |
|  |  | f_ Thermoanaerobacteraceae |
|  |  | f_ Dehalococcoidaceae |
|  |  | f_ Thermoleophilaceae |
|  |  | f_ Cystobacteraceae |
|  |  | f_ Desulfuromonadaceae |
|  |  | f_ Koribacteraceae |
|  |  | f_ Alteromonadaceae |
|  |  | f_ Bartonellaceae |
|  |  | f_ Coriobacteriaceae |
|  |  | f_ Thermolithobacteraceae |
|  |  | f_ Bradyrhizobiaceae |
|  |  | f_ Chlorobiaceae |
|  |  | f_ Segniliparaceae |
|  |  | f_ Enterococcaceae |
|  |  | f_ Microthrixaceae |
|  |  | f_ Nitrospinaceae |
|  |  | f_ Leuconostocaceae |
|  |  | f_ Sanguibacteraceae |
|  |  | f_ Propionibacteriaceae |
|  |  | f_ Solibacteraceae |
|  |  | f_ Syntrophobacteraceae |
|  |  | f_ Synergistaceae |
|  |  | f_ Thiotrichaceae |
|  |  | f_ Proteinivoraceae |
