# Supplementary material for: The ‘microbiome counterattack’: Insights on the soil and root‐associated microbiome in diverse chickpea and lentil genotypes after an erratic rainfall event
Source: Environ Microbiol Rep. 2023 May 24;15(6):459–83. doi: 10.1111/1758-2229.13167 (PMC10667653; doi:10.1111/1758-2229.13167)

**SUPPORTING INFORMATION**

Additional Supporting Information may be found in the online version of this article at the publisher’s website:

**Fig. S1.** Meteorological data. In the graph (a) the mm of rain for each day of the experimental period (from 04/06/2021 to 10/08/2021) are reported. In the graph (b) the minimum, maximum and average air temperature (°C) as well as the average relative humidity (%) are displayed for each day of the experimental period.

**Fig. S2.** Scatterplots of chickpea agronomic and eco-physiological measures. Chickpea genotype (An.Ca.1586, Nero Tolve, Pascià, Sultano) heights (cm) were measured in July and August 2021 (T0 and T1, respectively) (a-d). Chickpea genotype stomatal conductance (gsw) was measured at T0 and T1 (e-h). Irrigation treatments (RG) are reported in different colors as 100% water, not stressed (RG1), 50% water (RG2), 25% water (RG3).

**Fig. S3.** Scatterplots of lentil height. Lentil genotype (a: Colfiorito, b: Elsa, c: Eston, d: Itaca) heights (cm) were measured in July and August 2021 (T0 and T1, respectively). Irrigation treatments (RG) are reported in different colors as 100% water, not stressed (RG1), 50% water (RG2), 25% water (RG3).

**Fig. S4.** Heat tree and pie charts of chickpea bulk soil microbiome. The heat tree of the prokaryotic phyla present in chickpea bulk soil (a) and the pie charts (b, c, d) were generated with the web-based tool Microbiome Analyst (Dhariwal *et al*., 2017). The heat tree (a) depicts the hierarchical structure of phyla and the relative abundance of chickpea bulk soil microbial communities. The color gradient and the size of node, edge, and label are based on the log_2_ ratio of median abundance. The pie chart (b) shows the taxonomic abundance of chickpea bulk soil in both time points (T0 and T1). The pie charts (c, d) show the taxonomic abundance of chickpea bulk soil at T0 (July, c) and at T1 (August, d).

**Fig. S5.** Univariate analysis at feature level of chickpea bulk soil microbiome in July. The univariate analysis of the four chickpea genotypes (An.Ca.1586, Pascià, Sultano, Nero Tolve) was evaluated for the first time point in July (T0, a) and for the second time point in August (T1, b) with the web-based tool Microbiome Analyst (Dhariwal *et al*., 2017). In the graphs are shown the filtered count (left) and the Log-transformed count (right) for *Shinella fusca* (a) and for the genus *Mesorhizobium* (b).

**Fig. S6**. Univariate analysis at phylum level of chickpea bulk soil microbiome. The univariate analysis of the chickpea bulk soil was evaluated comparing the first (July, T0) and the second time point (August, T1) with the web-based tool Microbiome Analyst (Dhariwal *et al.,* 2017). In the graphs are shown the filtered count (left) and the Log-transformed count (right) for the phyla Firmicutes (a), Acidobacteria (b), Aquificae (c), Chlorobi (d) and Gemmatimonadetes (e).

**Fig. S7.** Univariate analysis at class level of chickpea bulk soil microbiome. The univariate analysis of the chickpea bulk soil was evaluated comparing the first (July, T0) and the second (August, T1) time point with the web-based tool Microbiome Analyst (Dhariwal *et al*., 2017). In the graphs are shown the filtered count (left) and the Log-transformed count (right) for the classes Deltaproteobacteria (a), Alphaproteobacteria (b), Dehalococcidia (c), Clostridia (d) Gloeobacterophycideae (e), Solibacteres (f), Aquificae (g) and Chlorobia (h).

**Fig. S8.** Univariate analysis at genus level of chickpea bulk soil microbiome. The univariate analysis of the chickpea bulk soil was evaluated comparing the first (July, T0) and the second (August, T1) time point with the web-based tool Microbiome Analyst (Dhariwal *et al*., 2017). In the graphs are shown the filtered count (left) and the Log-transformed count (right) for the genera *Sphingomonas* (a), *Solirubrobacter* (b), *Lysobacter* (c), *Arenimonas* (d), *Ilumatobacter* (e) and *Glycomyces* (f).

**Fig. S9.** Heat tree and pie charts of lentil bulk soil microbiome. The heat tree (a) depicts the hierarchical structure of phyla and the relative abundance of lentil bulk soil microbial communities. The color gradient and the size of node, edge, and label are based on the log_2_ ratio of median abundance. The pie chart (b) shows the taxonomic abundance of lentil bulk soil. The pie charts (c,d) show the taxonomic abundance of lentil bulk soil at T0 (c, July) and at T1 (d, August). The graphs were generated with the web-based tool Microbiome Analyst (Dhariwal *et al*., 2017).

**Fig. S10.** Univariate analysis at class and order level of lentil bulk soil microbiome. The univariate analysis of the lentil bulk soil was evaluated comparing the first (July, T0) and the second time point (August, T1) with the web-based tool Microbiome Analyst (Dhariwal *et al.,* 2017). In the graphs the filtered count (left) and the Log-transformed count (right) are shown for the class Alphaproteobacteria (a), and for the orders Sphingomonadales (b), Gaiellales (c), Rubrobacterales (d) and Clostridiales (e).

**Fig. S11.** Univariate analysis at family and genus level of lentil bulk soil microbiome. The univariate analysis of the lentil bulk soil was evaluated comparing the first (July, T0) and the second time point (August, T1) with the web-based tool Microbiome Analyst (Dhariwal *et al.,* 2017). In the graphs the filtered count (left) and the Log-transformed count (right) are shown for the families Sphingomonadaceae (a), Gaiellaceae (b), Geobacteraceae (c), Clostridiaceae (d) and Rubrobacteraceae (e) and for the genus Sphingomonas (f).

**Fig. S12**. Alpha and beta diversity of lentil bulk soil microbiome. The alpha diversity of the lentil bulk soil microbiome was evaluated at the first (July, T0, a) and at the second time point (August, T1, b) with the Chao1 index, and significant differences were evaluated with ANOVA. The beta diversity among the lentil genotypes (Colfiorito, Elsa, Eston and Itaca) was evaluated at T0 (c) with the Jaccard index, and significant differences were evaluated with PERMANOVA. The web-based tool Microbiome Analyst was used (Dhariwal *et al*., 2017).

**Fig. S13**. Lentil parcel showing disease symptoms. In the picture, a suffering lentil parcel of Itaca genotype is reported. Most of the plants in the parcel were dead, the others were suffering, displaying chlorotic leaves.

Fig. S1


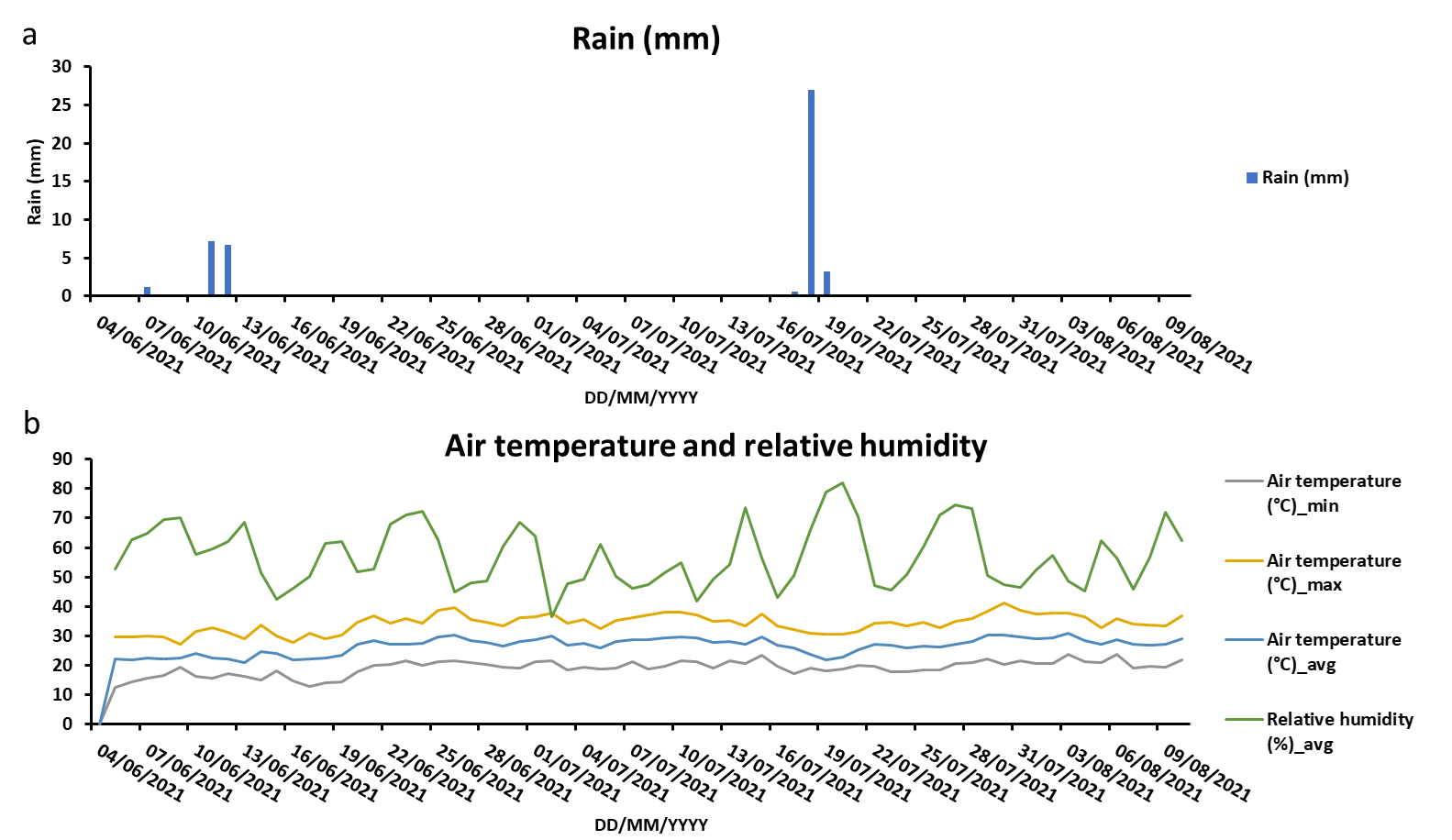


Fig. S2


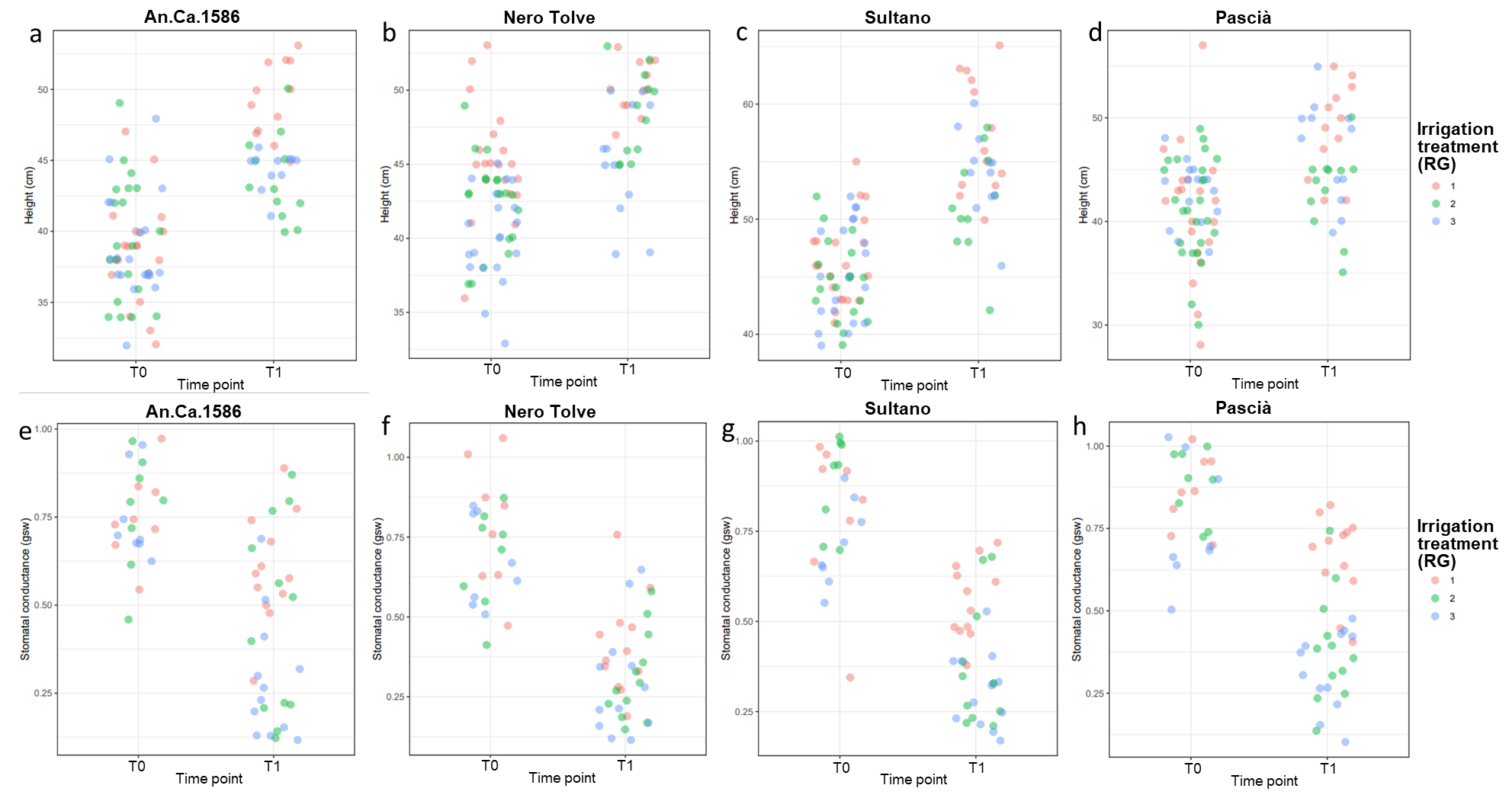


Fig. S3


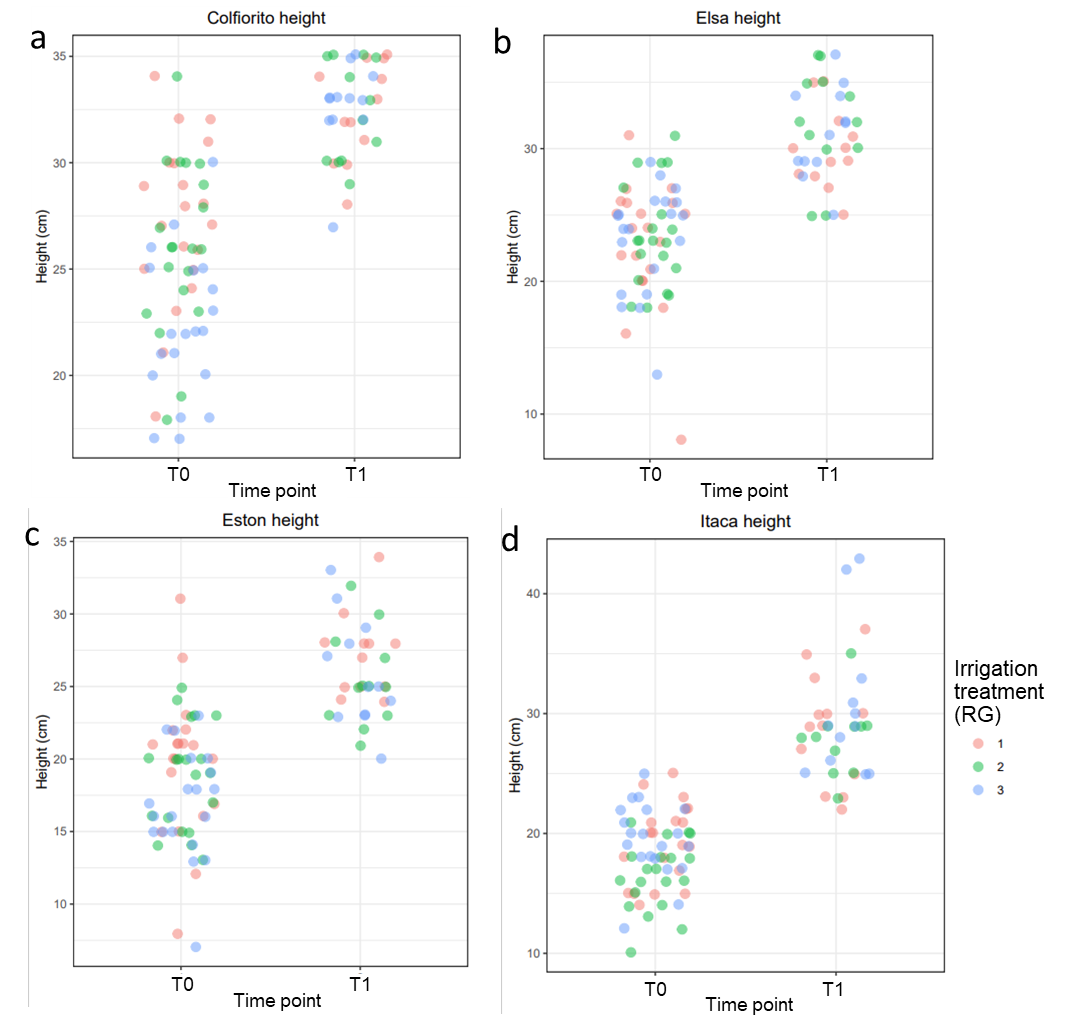


Fig. S4


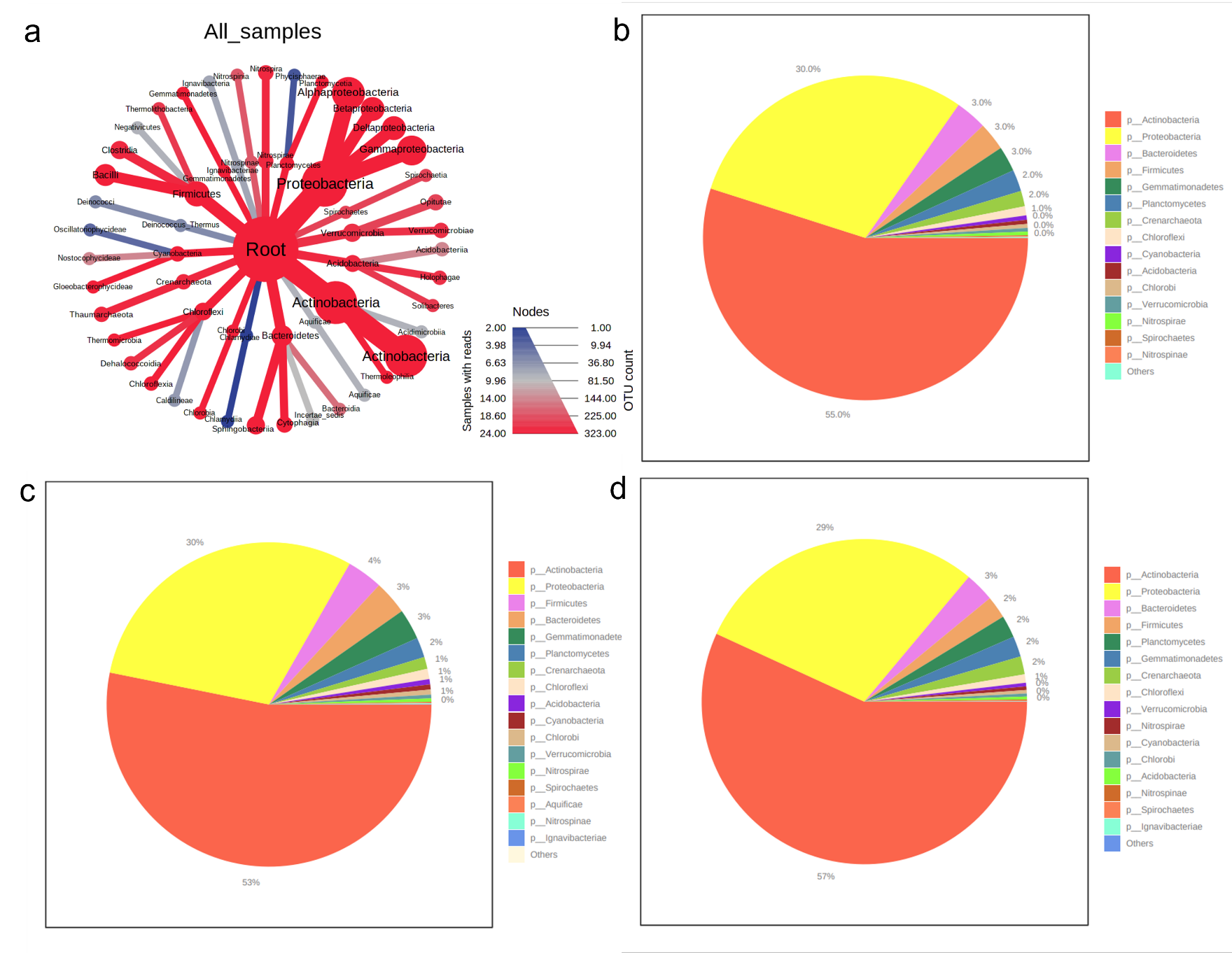


Fig. S5


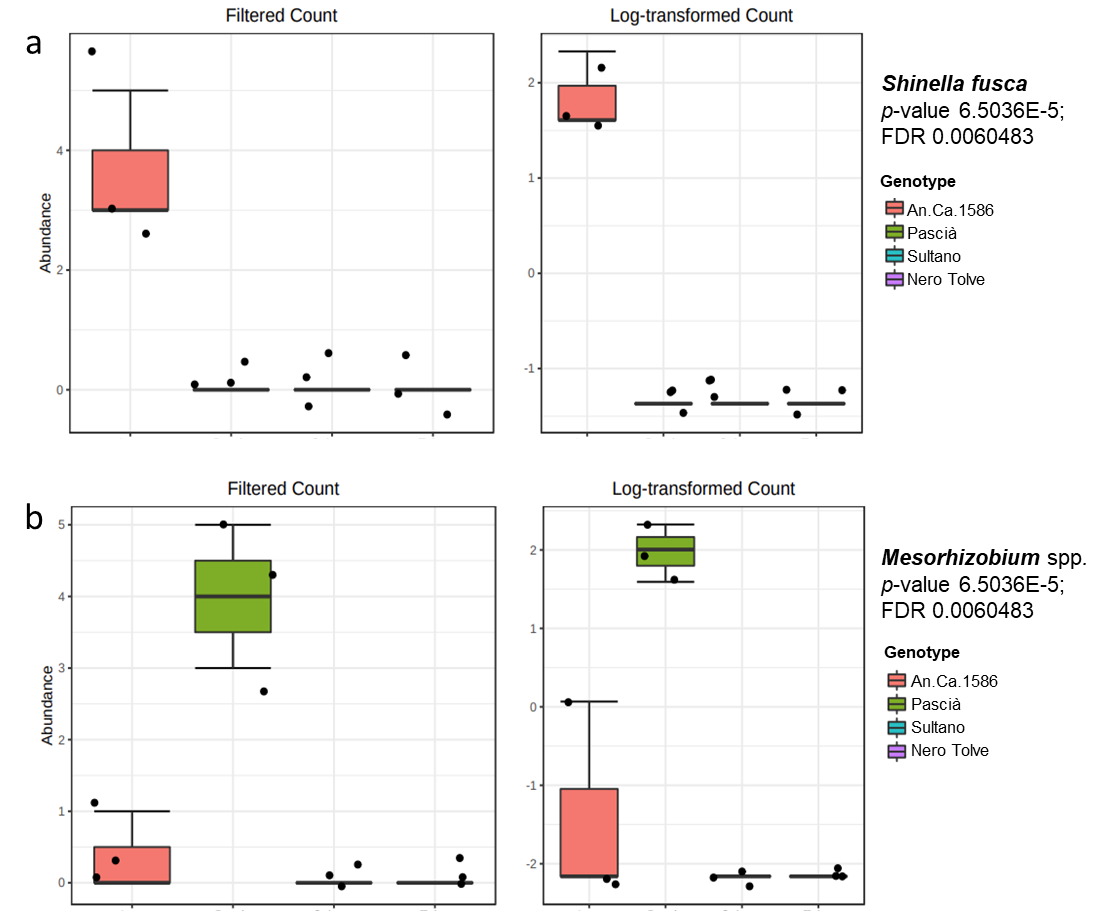


Fig. S6


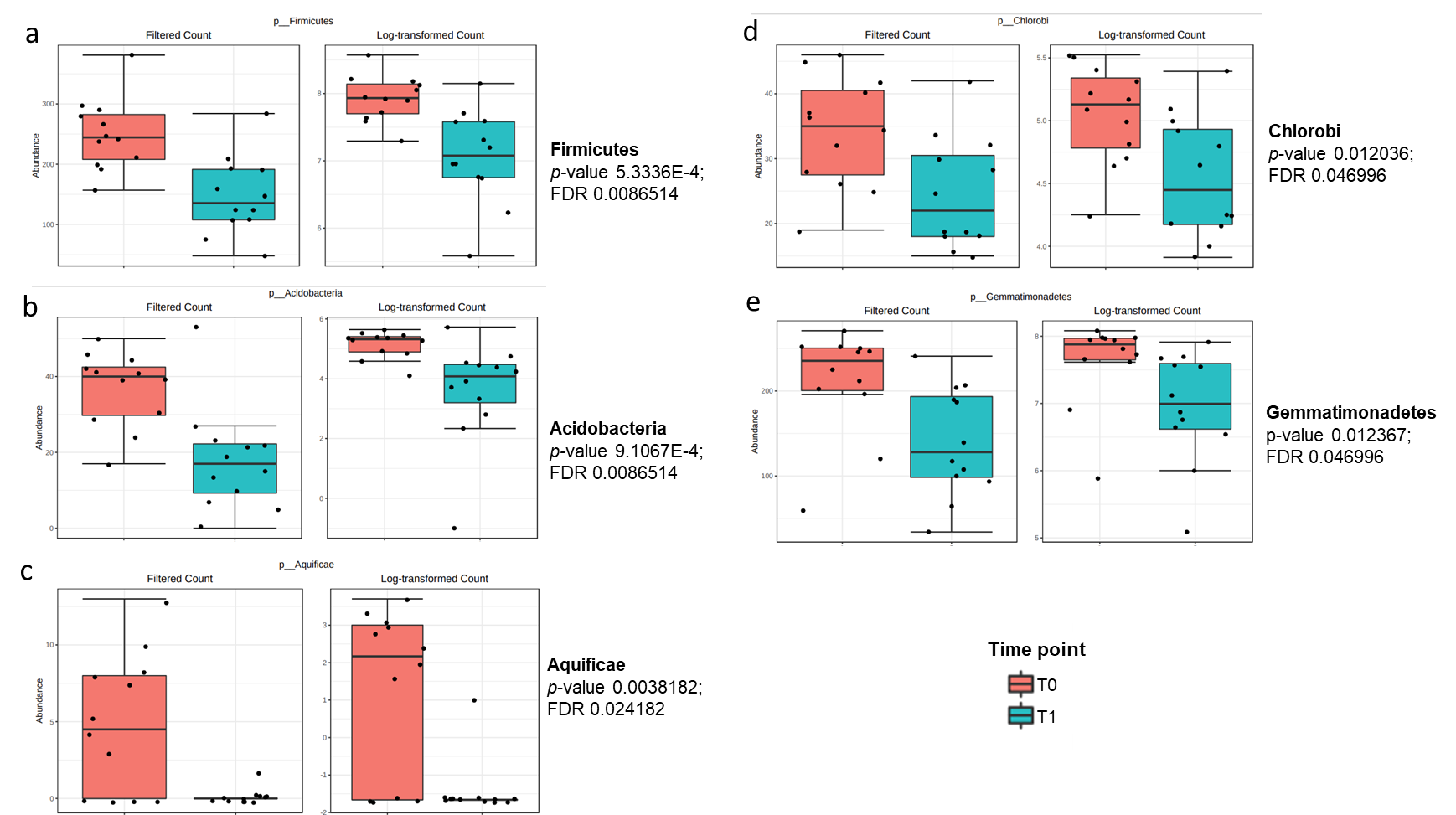


Fig. S7


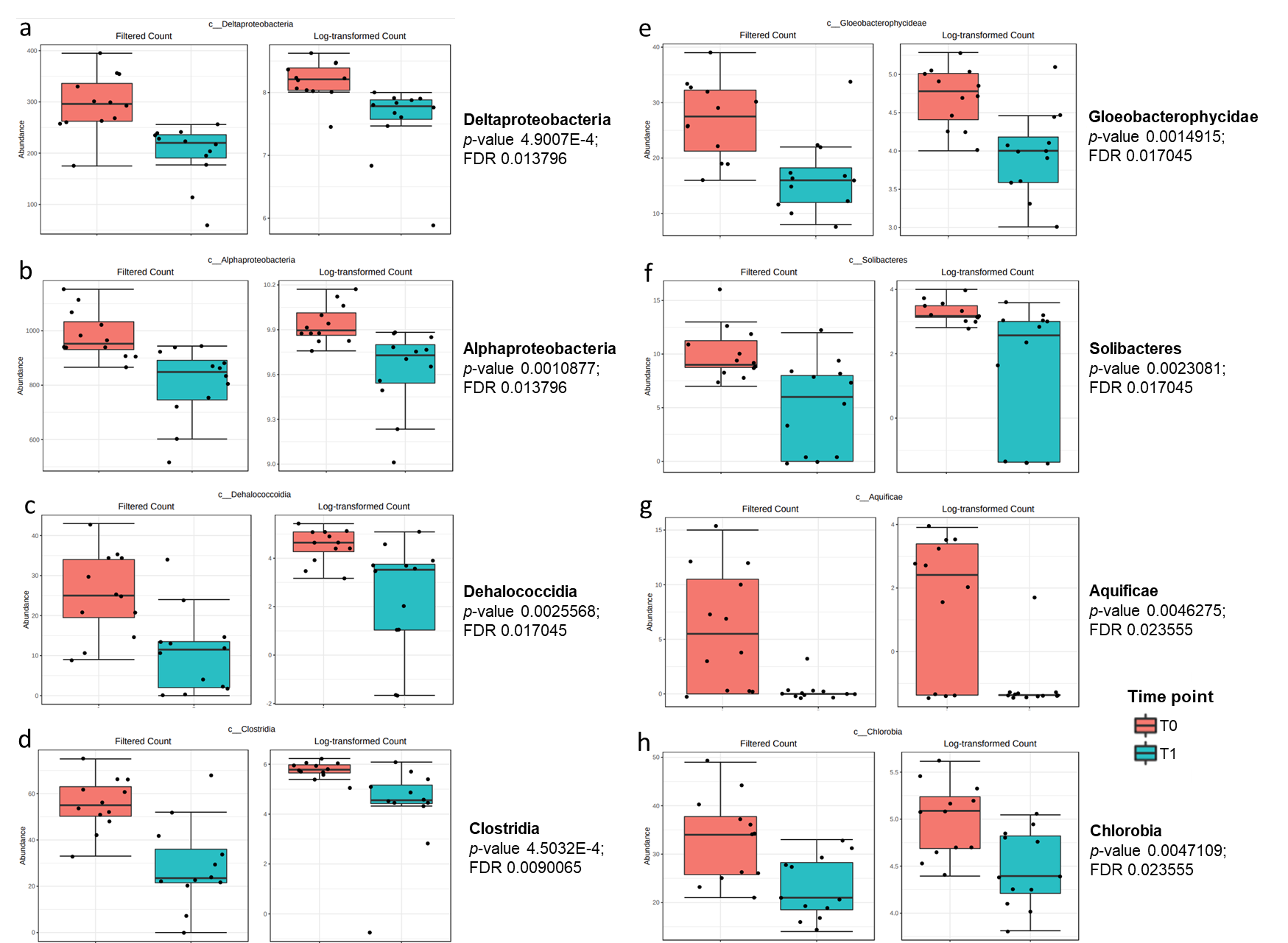


Fig. S8


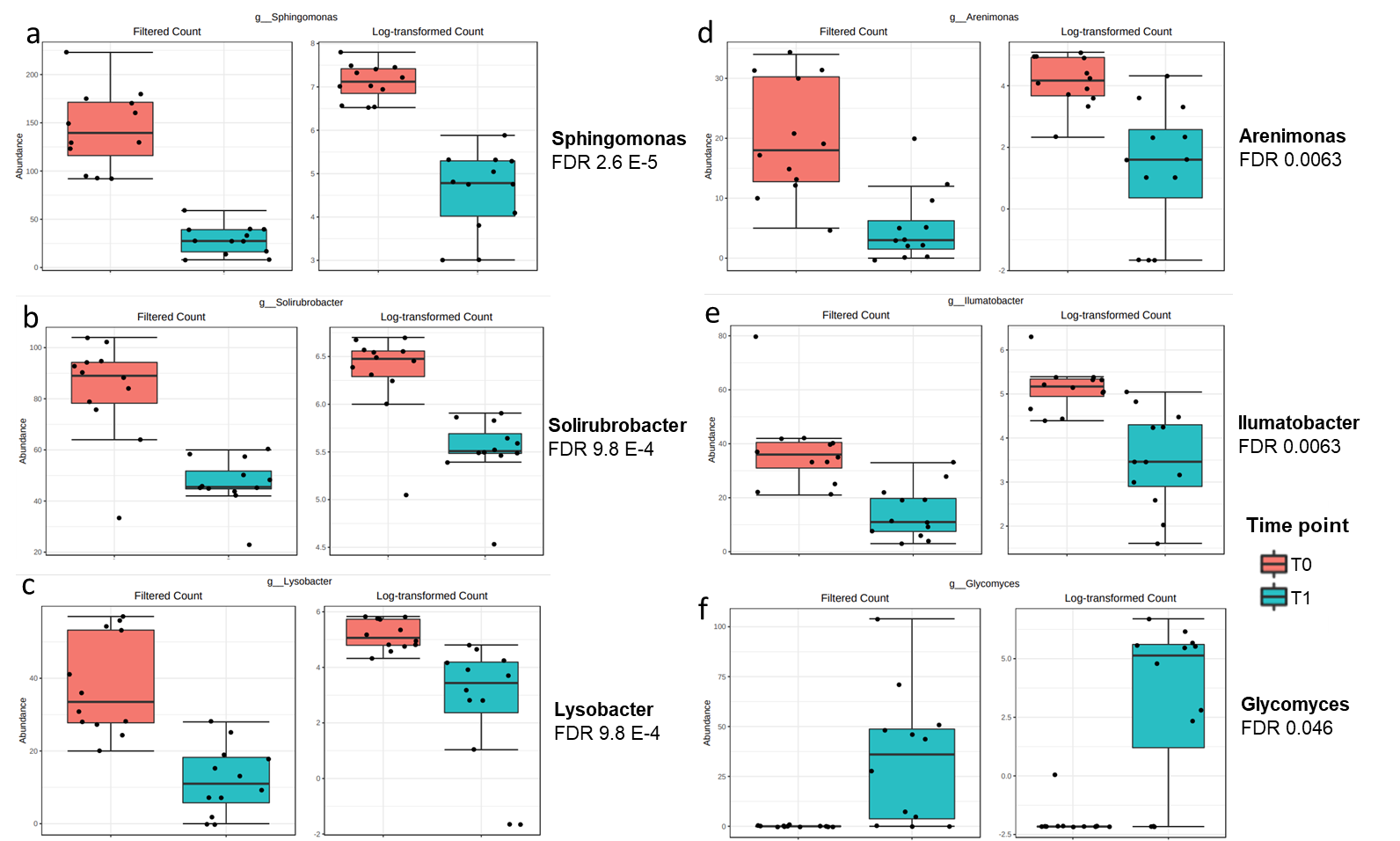


Fig. S9


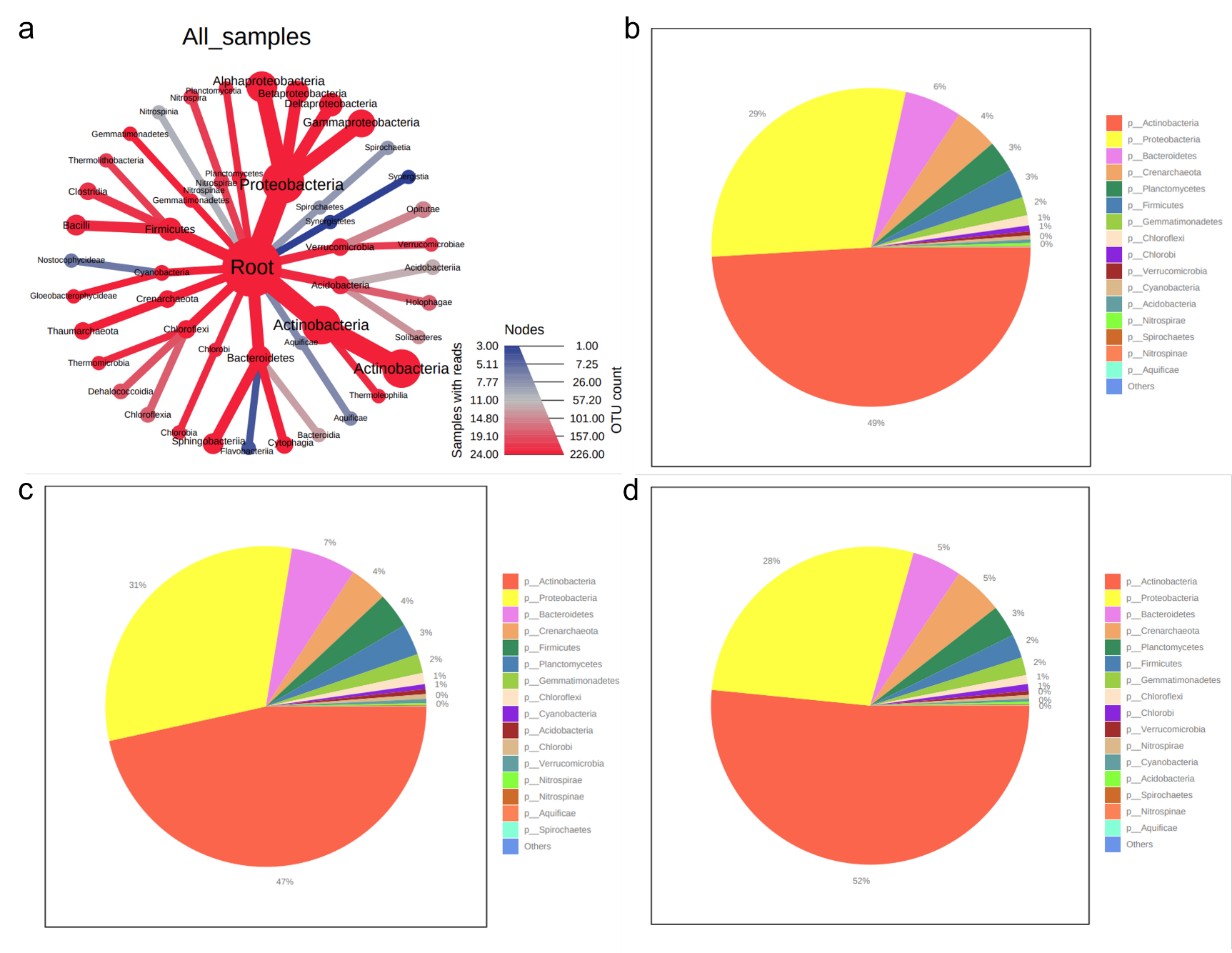


Fig. S10


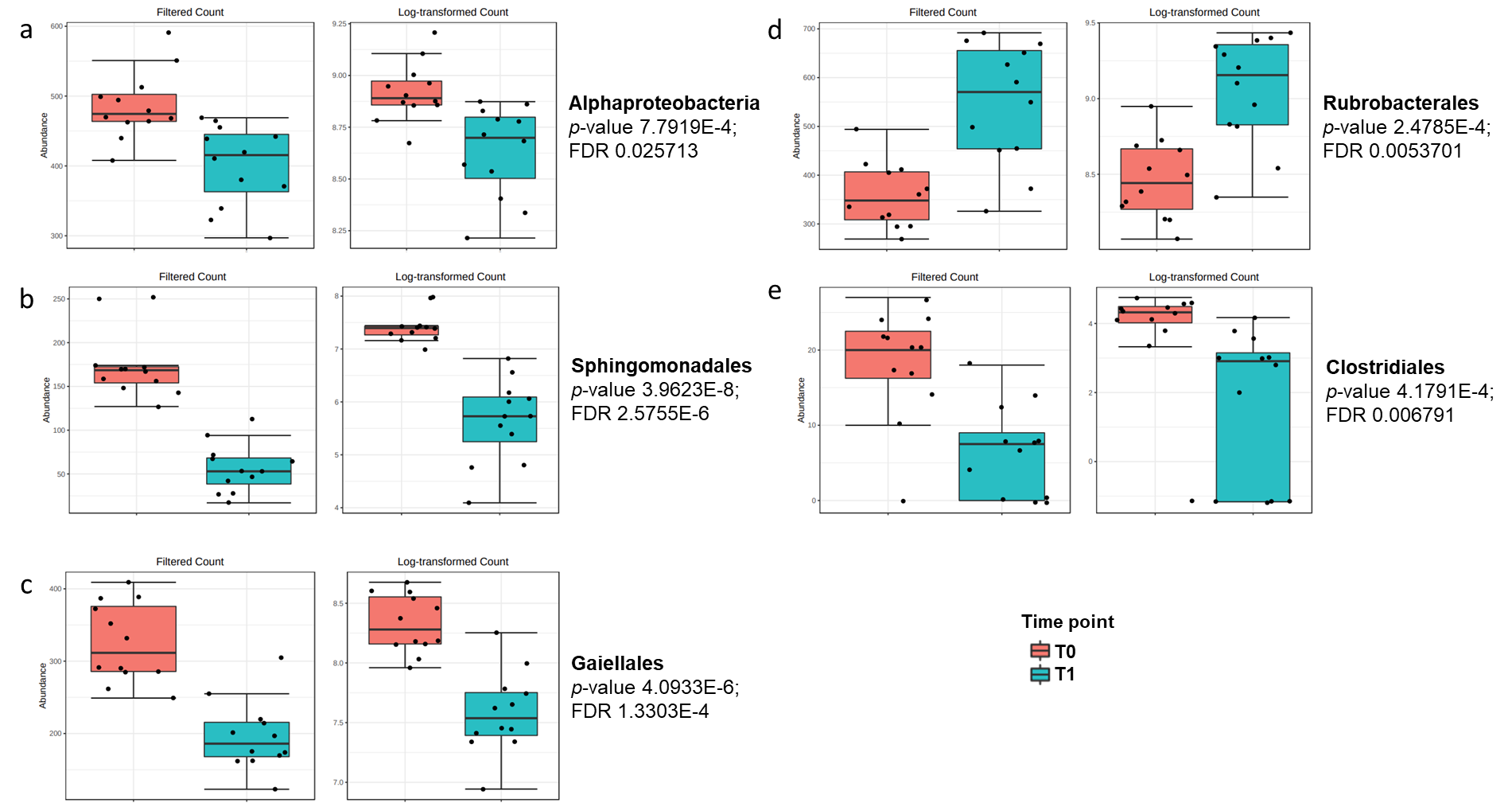


Fig S11


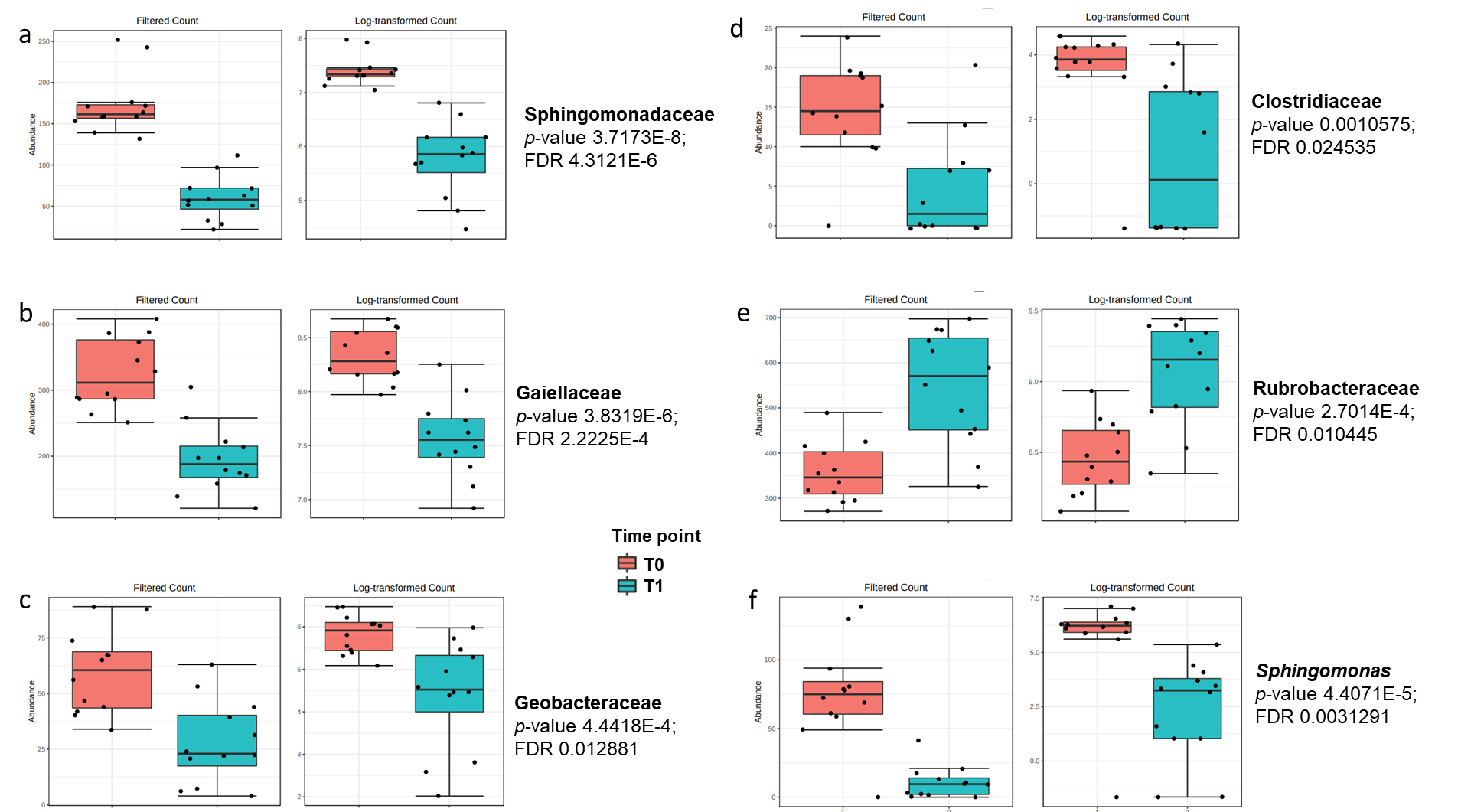


Fig. S12


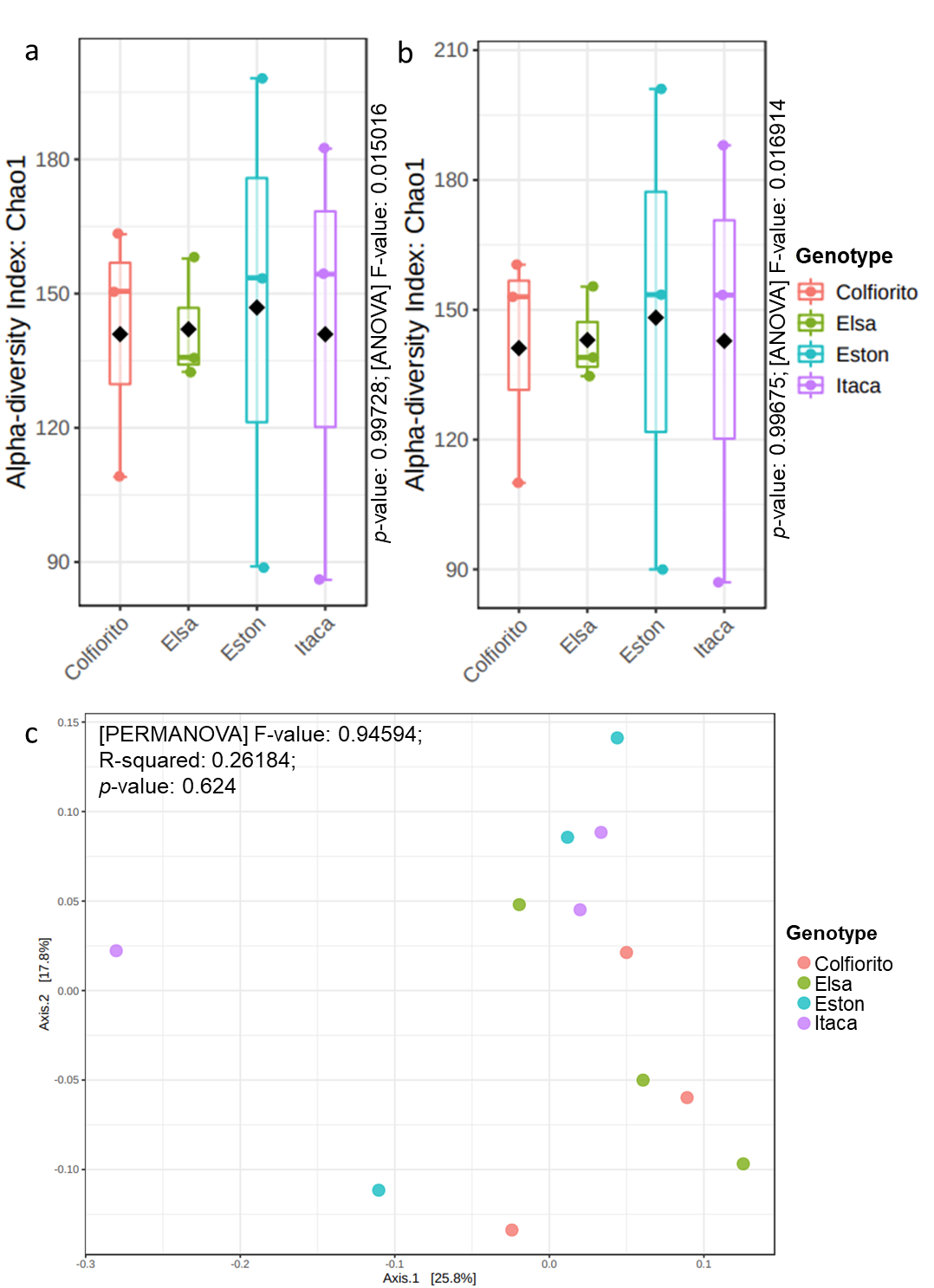


Fig. S13


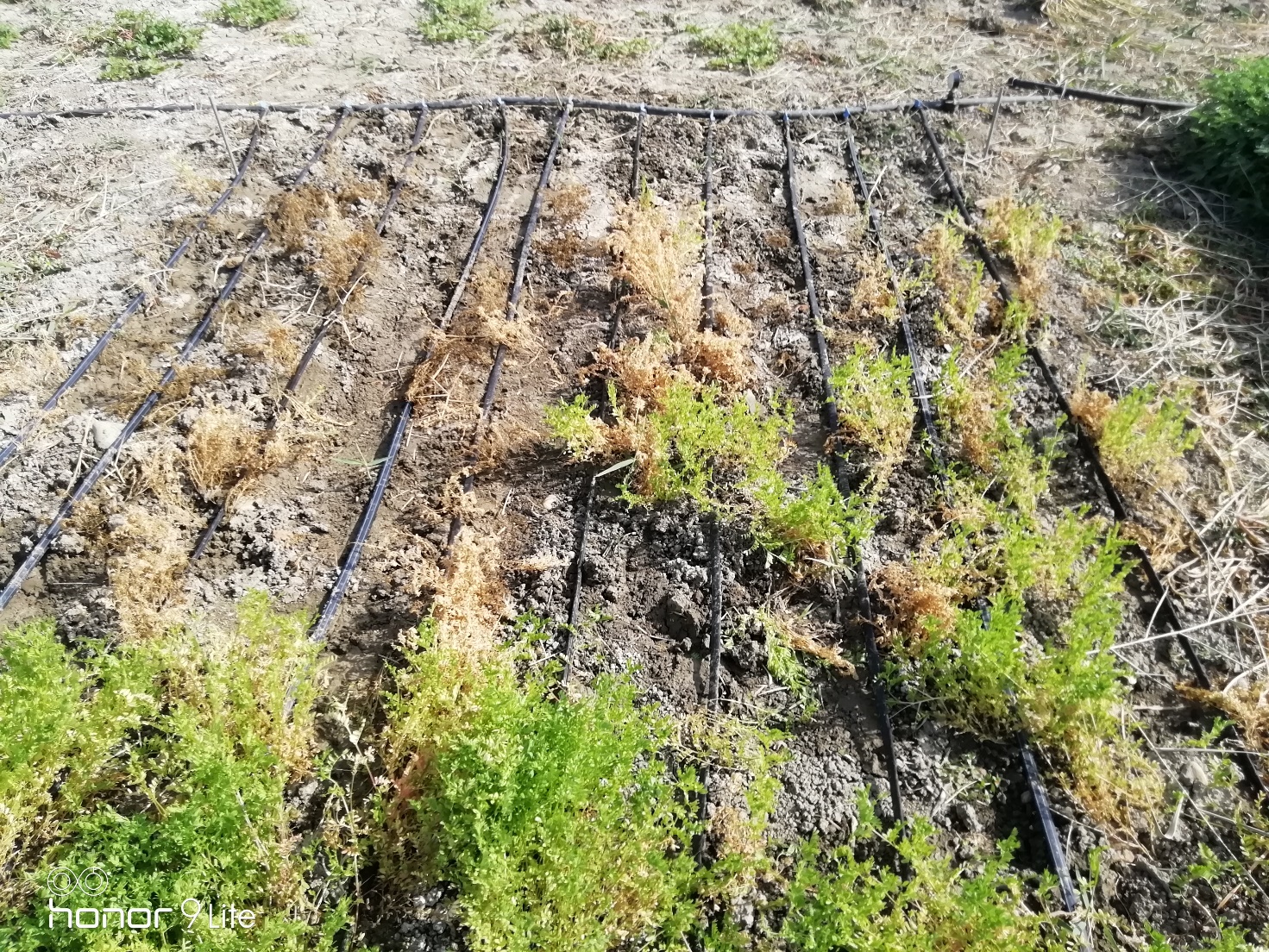

Supplement: Supplementary file 5 — Figure S1. Meteorological data. In the graph (a) the mm of rain for each day of the experimental period (from 04 June 2021 to 10 August 2021) are reported. In the graph (b) the minimum, maximum, and average air temperature (°C), as well as the average relative humidity (%), are displayed for each day of the experimental period. Figure S2. Scatterplots of chickpea agronomic and eco‐physiological measures. Chickpea genotype (An.Ca.1586, Nero Tolve, Pascià, Sultano) heights (cm) were measured in July and August 2021 (T0 and T1, respectively) (a)–(d). Chickpea genotype stomatal conductance (gsw) was measured at T0 and T1 (e)–(h). Irrigation treatments (RG) are reported in different colours as 100% water, not stressed (RG1), 50% water (RG2), and 25% water (RG3). Figure S3. Scatterplots of lentil height. Lentil genotype (a: Colfiorito, b: Elsa, c: Eston, d: Itaca) heights (cm) were measured in July and August 2021 (T0 and T1, respectively). Irrigation treatments (RG) are reported in different colours as 100% water, not stressed (RG1), 50% water (RG2), and 25% water (RG3). Figure S4. Heat tree and pie charts of chickpea bulk soil microbiome. The heat tree of the prokaryotic phyla present in chickpea bulk soil (a) and the pie charts (b)–(d) were generated with the web‐based tool Microbiome Analyst (Dhariwal et al., 2017). The heat tree (a) depicts the hierarchical structure of phyla and the relative abundance of chickpea bulk soil microbial communities. The colour gradient and the size of node, edge, and label are based on the log2 ratio of median abundance. The pie chart (b) shows the taxonomic abundance of chickpea bulk soil in both time points (T0 and T1). The pie charts (c), (d) show the taxonomic abundance of chickpea bulk soil at T0 (July, c) and at T1 (August, d). Figure S5. Univariate analysis at feature level of chickpea bulk soil microbiome in July. The univariate analysis of the four chickpea genotypes (An.Ca.1586, Pascià, Sultano, Nero Tolve) was evaluated for t [file EMI4-15-459-s004.docx]
